# Microglia Actively Remodel Adult Hippocampal Neurogenesis through the Phagocytosis Secretome

**DOI:** 10.1523/JNEUROSCI.0993-19.2019

**Published:** 2020-02-12

**Authors:** Irune Diaz-Aparicio, Iñaki Paris, Virginia Sierra-Torre, Ainhoa Plaza-Zabala, Noelia Rodríguez-Iglesias, Mar Márquez-Ropero, Sol Beccari, Paloma Huguet, Oihane Abiega, Elena Alberdi, Carlos Matute, Irantzu Bernales, Angela Schulz, Lilla Otrokocsi, Beata Sperlagh, Kaisa E. Happonen, Greg Lemke, Mirjana Maletic-Savatic, Jorge Valero, Amanda Sierra

**Affiliations:** ^1^Achucarro Basque Center for Neuroscience, Leioa, Bizkaia 48940, Spain,; ^2^University of the Basque Country UPV/EHU, Leioa, Bizkaia 48940, Spain,; ^3^Rudolf-Schönheimer-Institute of Biochemistry, Medical Faculty, University Leipzig 04109, Germany,; ^4^Laboratory of Molecular Pharmacology, Institute of Experimental Medicine, Hungarian Academy of Sciences, Budapest H-1083, Hungary,; ^5^Molecular Neurobiology Laboratory,; ^6^Immunobiology and Microbial Pathogenesis Laboratory, Salk Institute for Biological Studies, La Jolla, California 92037,; ^7^Jan and Dan Duncan Neurological Research Institute at Texas Children's Hospital, Houston, Texas 77030,; ^8^Department of Pediatrics and Neuroscience, Program in Developmental Biology, Baylor College of Medicine, Houston, Texas 77030, and; ^9^Ikerbasque Foundation, Bilbao, Bizkaia 48013, Spain

**Keywords:** adult neurogenesis, MerTK/Axl, microglia, P2Y12, phagocytosis, secretome

## Abstract

During adult hippocampal neurogenesis, most newborn cells undergo apoptosis and are rapidly phagocytosed by resident microglia to prevent the spillover of intracellular contents. Here, we propose that phagocytosis is not merely passive corpse removal but has an active role in maintaining neurogenesis. First, we found that neurogenesis was disrupted in male and female mice chronically deficient for two phagocytosis pathways: the purinergic receptor P2Y12, and the tyrosine kinases of the TAM family Mer tyrosine kinase (MerTK)/Axl. In contrast, neurogenesis was transiently increased in mice in which MerTK expression was conditionally downregulated. Next, we performed a transcriptomic analysis of the changes induced by phagocytosis in microglia *in vitro* and identified genes involved in metabolism, chromatin remodeling, and neurogenesis-related functions. Finally, we discovered that the secretome of phagocytic microglia limits the production of new neurons both *in vivo* and *in vitro*. Our data suggest that microglia act as a sensor of local cell death, modulating the balance between proliferation and survival in the neurogenic niche through the phagocytosis secretome, thereby supporting the long-term maintenance of adult hippocampal neurogenesis.

**SIGNIFICANCE STATEMENT** Microglia are the brain professional phagocytes and, in the adult hippocampal neurogenic niche, they remove newborn cells naturally undergoing apoptosis. Here we show that phagocytosis of apoptotic cells triggers a coordinated transcriptional program that alters their secretome, limiting neurogenesis both *in vivo* and *in vitro*. In addition, chronic phagocytosis disruption in mice deficient for receptors P2Y12 and MerTK/Axl reduces adult hippocampal neurogenesis. In contrast, inducible MerTK downregulation transiently increases neurogenesis, suggesting that microglial phagocytosis provides a negative feedback loop that is necessary for the long-term maintenance of adult hippocampal neurogenesis. Therefore, we speculate that the effects of promoting engulfment/degradation of cell debris may go beyond merely removing corpses to actively promoting regeneration in development, aging, and neurodegenerative diseases.

## Abstract (precis)

During adult hippocampal neurogenesis, most newborn cells undergo apoptosis and are rapidly phagocytosed by resident microglia to prevent the spillover of intracellular contents. Here, we propose that phagocytosis is not merely passive corpse removal but has an active role in maintaining neurogenesis.

## Introduction

Neurogenesis, or the formation of new neurons, is a complex process that extends throughout adulthood in specific regions of the mammalian brain. Here we focus on the subgranular zone of the hippocampus, whose radial neural stem cells (rNSCs) generate newborn granule cells in rodents (Ehninger and Kempermann, 2008) and humans (Moreno-Jimenez et al., 2019). Nowadays, newly generated neurons are strongly suggested to contribute to hippocampus-dependent learning and memory, among other functions (Deng et al., 2010). Multiple endogenous factors regulate the proliferation, survival, differentiation, and integration of the new neurons in the adult hippocampus. In the cellular niche, one key element is microglia, the resident macrophages of the nervous system that coordinate the brain inflammatory response. The detrimental effect of neuroinflammation on neurogenesis is well described, and is mediated by inflammatory cytokines such as interleukin (IL)-1β, tumor necrosis factor α (TNFα), and IL-6 (Ekdahl et al., 2003; Monje et al., 2003).

Microglia also beneficially affect neurogenesis, as they are capable of producing factors that modulate proliferation or survival of different cells within the neuronal lineage. *In vitro* studies demonstrate that cultured microglia promote differentiation of precursor cells (Aarum et al., 2003), whereas microglia-conditioned media enhances neuroblast production and neuronal survival (Morgan et al., 2004; Walton et al., 2006). Furthermore, microglia were suggested to inhibit the proliferation of hippocampal rNSCs, as their number inversely correlates with adult hippocampal neurogenesis (Gebara et al., 2013). Recently, experiments using diphtheria toxin-induced ablation of microglia propose that microglia are essential for neuroblast survival (Kreisel et al., 2019) but the mechanisms underlying the regulation of hippocampal neurogenesis by microglia are still unexplored both *in vitro* and especially *in vivo* (Sierra et al., 2014).

Here, we focus on another major role of microglia in the adult hippocampal neurogenic niche: the removal of apoptotic newborn cells through phagocytosis (Sierra et al., 2010). The majority of hippocampal newborn cells undergo apoptosis in the first few days of cells' life through adulthood (Beccari et al., 2017) and are immediately recognized and degraded by “unchallenged” microglia (Sierra et al., 2010). Microglia are the brain professional phagocytes compared with other cell types (Sierra et al., 2013) and prevent the release of toxic intracellular contents (Nagata et al., 2010), and thus, this process is essential to avoid alterations of the surrounding tissue.

In this study, we propose that microglial phagocytosis does not conclude with the physical elimination of apoptotic cells, but is followed by a coordinated transcriptional program that triggers the production of neurogenic modulatory factors, which directly contribute to the maintenance and correct regulation of the adult hippocampal neurogenic cascade. We have used constitutive and inducible knock-out (KO) mice to abolish two phagocytosis-related receptors: the purinergic receptor P2Y12 and the Mer tyrosine kinase (MerTK) of the TAM (Tyro, Axl, and Mer) family. We discovered that chronic phagocytosis deficiency disrupts neurogenesis but acute phagocytosis impairment only transiently increases neurogenesis. In addition, using a combined *in vitro* and *in vivo* based experimental strategy, we found that the secretome of phagocytic microglia limits the production of new neurons to maintain the homeostasis of the adult hippocampal neurogenic niche.

## Materials and Methods

### 

#### 

##### Mice.

All experiments were performed in fms-EGFP (MacGreen) mice, except where indicated, in which all microglia express the fluorescent reporter (Sasmono et al., 2003; Sierra et al., 2007). KO mice were provided by Beata Sperlagh, Institute of Experimental Medicine (P2Y12 KO) and Greg Lemke, Salk Institute (MerTK/Axl KO). Microglial-specific, inducible MerTK/Axl mice were generated using *Cx3cr1^CreER^* (Parkhurst et al., 2013) and *Mertk^fl/fl^* (Fourgeaud et al., 2016), described previously. To induce deletion of the *Mertk^fl/fl^* allele in *Cx3cr1*^*CreER*/+^*Mertk^fl/fl^* mice, two doses of tamoxifen dissolved in corn oil (75 mg/kg) or corresponding volume of corn oil alone (control mice) were administered intraperitoneally at postnatal days (P)21 and P23. All mice used were in a C57BL/6 background. Mice were housed in 12 h light/dark cycle with *ad libitum* access to food and water. Mice received a single dose of 5-bromo-2′-deoxyuridine (BrdU; 100–150 mg/kg) at P28. At 24 h or 28 d after BrdU injection, mice were anesthetized with a mixture of ketamine and xylazine (100 mg/kg and 10 mg/kg, respectively), perfused with 20 U/ml heparin in PBS followed by 4% PFA in PBS. Brains were collected, immersion fixed for 4 h in 4% PFA in PBS, and stored in 30% sucrose, 30% ethylene glycol at −20°C until analysis. All procedures followed the European Directive 2010/63/EU and NIH guidelines, and were approved by the Ethics Committees of the University of the Basque Country EHU/UPV (Leioa, Spain; CEBA/205/2011, CEBA/206/2011, CEIAB/82/2011, CEIAB/105/2012).

##### SH-SY5Y cell line.

SH-SY5Y (American Type Culture Collection), a human neuroblastoma cell line derived from the bone marrow of 4-year-old female was used for phagocytic assay experiments. SH-SY5Y cells were grown as an adherent culture in non-coated culture flasks covered with 10–15 ml of medium. The medium consisted of DMEM (Invitrogen), supplemented with 10% fetal bovine serum (FBS) and 1% antibiotic/antimycotic (all from Invitrogen). When confluency was reached, cells were trypsinized and re-plated at 1:4.

##### BV2 cell line.

BV2 (Interlab Cell Line Collection San Martino-Instituto Scientifico Tumori-Instituto Nazionale per la Ricerca sul Cancro), a cell line derived from raf/myc-immortalized murine neonatal microglia was used to obtain LPS-induced conditioned media. BV2 cells were grown as an adherent culture in non-coated culture flasks covered with 10–15 ml of medium. The medium consisted of DMEM (Invitrogen), supplemented with 10% FBS and 1% antibiotic/antimycotic (all from Invitrogen). When confluency was reached, cells were trypsinized and replated at 1:4.

##### Primary microglia cultures.

Primary microglia cultures were performed as previously described (Abiega et al., 2016; Beccari et al., 2018). P0–P1 fms-EGFP mice pup brains were extracted and the meninges were peeled off. The olfactory bulb and cerebellum were discarded and the rest of the brain was then mechanically homogenized by careful pipetting and enzymatically digested with papain (20 U/ml; Sigma-Aldrich), a cysteine protease enzyme, and DNase (150 U/μl; Invitrogen) for 15 min at 37°C. The resulting cell suspension was then filtered through a 40 μm nylon cell strainer (Fisher) and transferred to a 50 ml Falcon tube quenched by 5 ml of 20% FBS (Invitrogen) in HBSS. Afterward, the cell suspension was centrifuged at 200 × *g* for 5 min, the pellet was resuspended in 1 ml DMEM (Invitrogen) supplemented with 10% FBS and 1% antibiotic/antimycotic (Invitrogen), and seeded in T75 poly-l-lysine-coated (15 μl/ml; Sigma-Aldrich) culture flasks at a density of two brains per flask. Medium was changed the day after and then every 3–4 d, always enriched with granulocyte-macrophage colony stimulating factor (5 ng/ml GM-CSF; Sigma-Aldrich), which promotes microglial proliferation. After confluence (at 37°C, 5% CO_2_ for ∼14 d), microglia cells were harvested by shaking at 100–150 rpm, 37°C, 4 h. Isolated cells were counted and plated at a density of 80,000 cells/well on poly-l-lysine-coated glass coverslips in 24-well plates for immunofluorescence purposes or 1,000,000 cell/dish on coated Petri dishes for real-time quantitative PCR (qPCR). Microglia were allowed to settle for at least 24 h before any experiment.

##### NPC culture.

Neurosphere cultures were performed as previously described (Babu et al., 2011) with some modifications. Briefly, P0–P1 fms-EGFP pups were decapitated and the brains extracted and placed in cold HBSS. The homogenization process was performed as detailed in the Primary microglia cultures section, except that no FBS was used in any step to avoid undesired neurosphere adhesion and differentiation. Afterward, the cell suspension was centrifuged at 200 × *g* for 5 min, the pellet was resuspended in 1 ml DMEM/F12 with GlutaMAX (Invitrogen) supplemented with 1% penicillin/streptomycin, 1% B27, EGF (12.5 ng/ml), FGF-2 (5 ng/ml; Xapelli et al., 2013). Cells were plated on uncoated Petri dishes (P60); each brain was plated in four Petri dishes with supplemented DMEM/F12. After 6 d, neurospheres were then disaggregated into a single-cell suspension of neuroprogenitor cells (NPCs) using NeuroCult chemical dissociation kit following the manufacturer's instructions and each Petri dish was plated in two 6-multiwell plates. To maintain replicability through the experiments, neurospheres were frozen until their use at −80°C in 15% DMSO after the first passage.

##### *In vitro* phagocytosis assay.

The protocol was detailed previously (Beccari et al., 2018). In brief, microglia were allowed to rest and settle for at least 24 h before phagocytosis experiments. Phagocytosis experiments were performed in DMEM +10% FBS to ensure the presence of complement molecules, which are related to microglial phagocytosis *in vivo* (Diaz-Aparicio and Sierra, 2019) and whose presence determines the immunomodulatory outcome of phagocytosis (Fraser et al., 2010). Primary microglia cells were fed for different time points with SH-SY5Y. The cell line was previously labeled with the membrane marker CM-DiI (5 μm; 10 min at 37°C, 15 min at 4°C; Invitrogen) and treated with staurosporine (STP; 3 μm, 4 h; Sigma-Aldrich) to induce apoptosis. Only the floating dead-cell fraction was collected from the supernatant and added to the primary microglia cultures in a proportion of ∼1:1. Apoptotic cells were visualized and quantified by trypan blue in a Neubauer chamber. Because cell membrane integrity is still maintained in early induced apoptotic cells, cells not labeled with trypan blue were considered apoptotic. The media of naive and phagocytic (24 h) microglia was immediately stored at −80°C until its use as conditioned media for NPCs.

In some experiments, control and phagocytic microglia were treated with LPS. Three different LPS paradigms were used. In the low LPS concentration paradigm, media was removed and fresh medium with 150 ng/ml LPS or vehicle (PBS) was added for 18 h to primary microglia (Fraser et al., 2010). In the high LPS concentration paradigm, medium was removed and fresh medium with 1 μg/ml LPS or vehicle (PBS) was added for 24 h to primary or BV2 cells (Monje et al., 2003). To control for LPS presence in the phagocytic media, a third paradigm was performed in which primary microglia was treated with 1 μg/ml LPS or vehicle (PBS) for 6 h, then media was changed into fresh media for another 18 h. All supernatants were collected and stored at −80°C until its use as conditioned media for NPCs, and all of them were filter-sterilized before adding to the NPC culture.

##### NPC proliferation and differentiation.

Neurospheres of Passage 1 were thawed and expanded for 1 week before the experiment in proliferative conditions (2 passages were performed in total).The day of the experiment Passage 3 neurospheres were dissociated into NPCs, cells were counted and plated at a 80,000 cells/well density on poly-l-lysine-coated glass coverslips in 24-well plates in supplemented (Penicillin/streptomycin, B27, EGF, and FGF2) DMEM/F12. NPCs were allowed to proliferate for 48 h (Babu et al., 2011) and then washed with PBS before treatment with conditioned media (CM) from control or phagocytic (24 h) microglia. For the experimental group, DMEM was also added as a control because it is the media in which microglia were grown. NPCs were then fixed with 4% PFA for 10 min at 3 d, and 5 d of differentiation. For multipotency experiments, NPCs treated for 3 d with CM (control, 24 h phagocytosis or DMEM) were transferred back to DMEM/F12 (without trophic factors) medium and were allowed to differentiate for 5 and 9 d. For late survival and differentiation assay, after the 48 h of proliferation, NPCs were allowed to differentiate in DMEM/F12 (no trophic factors) for 10 d and then were treated with CM from control or phagocytic (24 h) microglia or DMEM for another 3 and 5 d.

##### Calcium imaging.

Intracellular calcium imaging experiments were performed as described previously (Alberdi et al., 2013). CM-treated NPCs were incubated and loaded with 5 μm Fura-2 AM (Invitrogen) for 30 min at 37°C and then washed in HBSS containing 20 mm HEPES, pH 7.4, 10 mm glucose, and 2 mm CaCl_2_ for 10 min at room temperature. The perfusion chamber was assembled on the platform of an inverted epifluorescence microscope (Zeiss Axiovert 35) equipped with a 150-W xenon lamp Polychrome IV (TILL Photonics) and a Plan Neofluar 40× oil-immersion objective (Zeiss). NPCs were treated with 50 mm KCl, 10 μm AMPA, 1 mm ATP, and 100 μm histamine, sequentially. Cells were allowed to recover their baseline before adding the next compound. Cells were visualized with a digital black/white CCD camera (ORCA; Hamamatsu Photonics). Intracellular calcium signaling responses were calculated as the proportion of different cell phenotypes responding to the different stimuli. The baseline was calculated as the mean of the first 60 s of recording for each cell. Only peaks that increase or decrease three times the SEM of the baseline were considered as a significant response.

##### FACS sorting.

Microglia cells were isolated from brains as described previously (Sierra et al., 2007; Abiega et al., 2016). The corresponding tissues from fms-EGFP mice were dissected and placed in enzymatic solution (in mm: 116 NaCl, 5.4 KCl, 26 NaHCO_3_, 1 NaH_2_PO_4_, 1.5 CaCl_2_, 1 MgSO_4_, 0.5 EDTA, 25 glucose, 1 l-cysteine) with papain (20 U/ml) and DNase I (150 U/μl; Invitrogen) for digestion at 37°C for 15 min. The homogenization process was also helped by careful pipetting. After homogenization, tissue clogs were removed by filtering the cell suspension through a 40 μm nylon strainer to a 50 ml Falcon tube quenched by 5 ml of 20% FBS in HBSS. For further enrichment of microglia, myelin was removed by using Percoll gradients. For this purpose, cells were centrifuged at 200 × *g* for 5 min and resuspended in a 20% solution of isotonic percoll (SIP; 20% in HBSS), obtained from a previous stock of SIP (9 parts Percoll per 1 part PBS 10×). Then, each sample was layered with HBSS poured very slowly by fire-polished pipettes. Afterward, gradients were centrifuged for 20 min at 200 × *g* with minimum acceleration and no brake so the interphase was not disrupted. Then the interphase was removed, cells were washed in HBSS by centrifuging at 200 × *g* for 5 min and pellet was resuspended in 500 μl of sorting buffer (25 mm HEPES, 5 mm EDTA, 1% BSA, in HBSS). Microglia cell sorting was performed by FACS Jazz (BD Biosciences), in which the population of green fluorescent cells was selected, collected in Lysis Buffer (Qiagen) containing 0.7% β-mercaptoethanol and stored at −80°C until processing.

##### Administration of microglia CM *in vivo*.

CM from control and phagocytic (Ph24h) microglia was administrated via osmotic pumps for 6-d to 2-month-old fms-EGFP mice. Briefly, osmotic pump (flow rate 1 μl/h; Model 2001, Alzet) and infusion catheter tubes (Alzet) were filled with the conditioned media (200 μl) and connected. Pumps were incubated overnight at 37°C in PBS before the surgery. Mice were anesthetized with ketamine/xylazine (10/1 mg/kg) and received a single dose of the analgesic buprenorphine (1 mg/kg) subcutaneously. The infusion cannulae were inserted at anteroposterior: −1.7 mm, laterolateral: −1.6 mm, and dorsoventral: −1.9 mm from bregma. The injection site did not reach nor damage the DG in any of the mice included in the study. Afterward, a surface of dental cement was created from the cannulae to the screw to avoid any unwanted removal of the cannulae. Osmotic pumps were inserted inside the skin of the mice. After 6 d, mice were intraperitoneally injected with BrdU (150 mg/kg, single injection), and transcardially perfused 2 h later to assess proliferation. For differentiation experiments, CM-containing osmotic pumps were inserted for 6-d to 2-month-old fms-EGFP mice. Pumps were removed at 6 d and afterward, a single intraperitoneal injection of BrdU (150 mg/kg) was administered, and mice were killed 28 d later.

##### Gene expression arrays.

Gene arrays analysis was performed following the recommendations of the MIAME (Minimum Information About a Microarray Experiment) consortium (Brazma et al., 2001). Cell samples from control, Ph3h, and Ph24h microglia (*n* = 3 independent experiments) were lysed and kept at −80°C until processing. Total RNA was isolated using PureLink RNA Mini kit (Ambion), following the manufacturer's instructions. RNA amount was quantified in a UV/VIS NanoDrop 1000 spectrophotometer (ThermoFisher Scientific), and its integrity was analyzed with Lab-chip technology in an Agilent 2100 Bioanalyzer in combination with Agilent RNA 6000 Nano Chips. Eukaryote Total RNA Nano Assay was used as type of test. In all samples, RIN > 9.3, and 28S/18S > 1.3 values were obtained. Sample labeling, hybridization, and scanning gene expression profiling were performed at the Gene Expression Unit of Genomics Core Facility of the University of the Basque Country UPV/EHU.

One-color microarray-based gene expression analysis was performed following the One-Color (p/n5190–2305) protocol from Agilent Technologies (Low Input Quick Amp Labeling kit) for the labeling of the samples. First, 50 ng of total RNA were retrotranscribed with the AffinityScript Reverse enzyme Transcriptase (AffinityScript RT), a thermostable modified enzyme derived from Moloney murine leukemia virus retrotranscriptase, using promoter-coupled T7 Oligo dT primers. The double-stranded cDNA synthesized by AffinityScript RT was transcribed *in vitro* by the T7 RNA pol in the presence of Cy3-CTP to generate labeled and amplified cRNA. The labeled samples were purified with columns of RNeasy Mini kit (Qiagen). Subsequently the labeled samples were quantified in the NanoDrop ND-1000 to determine the performance of the specific activity of the fluorochromes after labeling. All the hybridized samples met the following minimum requirements: yield > 0.825 μg per reaction and cyanine 3-specific activity > 6 pmol/μg.

For the hybridization, 600 ng of labeled cRNA were fragmented and cohybridized to SurePrint G3 Mouse GE 8X60K Microarray Design ID: 028005. Each array/slide contained 8 identical subarrays of >60,000 60-mer oligonucleotides of high resolution and performance. It contained probes for 55,681 sequences or transcripts (biological features or non-control features). Several of these biological probes were replicated 10 times for the calculations and quality control measurements (QCMetrics) of the microarrays. It also contained probes for internal positive controls (spike-ins), which were added to the RNA sample before labeling and were used for evaluation and verification of the microarray processing. Manual washing method was performed following Agilent's recommendations to prevent ozone-related problems.

Slides were scanned on a G2565CA Microarray DNA Scanner from Agilent Technologies with a resolution of 3 μm and a Tiff image size of 20bit, using the Scan software v8.5.1 with default settings (Scan profile Agilent, G3_GX_1color). The scanned TIFF images were processed and the fluorescence of the probes quantified using Agilent Feature Extraction software 10.7.3.1. Feature Extraction protocol for data extraction: GE1_107_Sep09; Design File: 28005_D_F_20140728. Software extracts information of the raw fluorescence signal (mean signal) for the fluorochrome or channel (Cy3: green channel) from the spot containing the probes (positive and negative controls and no controls or biological feature) and the background, obtained from the negative controls (which contains sequences for which no hybridization is expected, nonspecific binding indicators).

Default parameters (Agilent Feature Extraction software 10.7.3.1) for one-color gene expression microarrays were used for flagging of non-uniform features, population outliers for replicated probes, and features with no significant intensities in Cy3 channel. Agilent Feature Extraction raw data were processed with software GeneSpring GX 13.0 (Agilent Technologies). Probes not present in any sample were filtered out. A list of the filtered 36,665 probes was used in the statistical analysis.

##### Tissue or cultured cells RNA isolation and retrotranscription.

The corresponding tissue (P8 hippocampi for positive PCR controls) was rapidly isolated immediately under tribromoethanol overdose, and stored at −80°C. Tissue was disrupted with a roto-stator homogenizer with Lysis Buffer (Qiagen) containing 0.7% β-mercaptoethanol and stored at −80°C until processed. Cultured cells (>500,000 cells) were lysed and stored at −80°C until processed. Total RNA was isolated using Qiagen RNeasy Mini Kit (Qiagen), following the manufacturer's instructions, including a DNase treatment step to eliminate genomic DNA residues. RNA was quantified in a NanoDrop 2000, and 1.5 μg were retrotranscribed using random hexamers (Invitrogen) and Superscript III Reverse Transcriptase kit (Invitrogen), following the manufacturer's instructions in a Veriti Thermal Cycler (Applied Biosystems).

##### FACS-sorted cells RNA isolation and retrotranscription.

RNA from FACS-sorted microglia (<500,000 cells) was isolated by RNeasy Plus micro kit (Qiagen) according to the manufacturer's instructions, and the RNA was retrotranscribed using an iScript Advanced cDNA Synthesis Kit (Bio-Rad) following the manufacturer's instructions in a Veriti Thermal Cycler (Applied Biosystems).

##### Real-time qPCR.

Real-time qPCR was performed following MIQE guidelines (Minimal Information for Publication of Quantitative Real Time Experiments; Bustin, 2010). Three replica of 1.5 μl of a 1:3 dilution of cDNA were amplified using Power SYBR Green (Bio-Rad) for tissue or cell culture experiments or SsoFast EvaGreen Supermix (Bio-Rad) for FACS-sorted microglia experiments in a CFX96 Touch Real-Time PCR Detection System (Bio-Rad). The amplification protocol for both enzymes was 3 min 95°C, and 40 cycles of 10 s at 95°C, 30 s at 60°C.

##### Primers.

Primers were designed to amplify exon–exon junctions using PrimerBlast (NIH) to avoid amplification of contaminating genomic DNA, and their specificity was assessed using melting curves and electrophoresis in 2% agarose gels. Primer sequences are listed in Table 1. For each set of primers, the amplification efficiency was calculated using the software LinRegPCR (Ramakers et al., 2003) or standard curve of 1:2 consecutive dilutions, and was used to calculate the relative amount using the following formula:

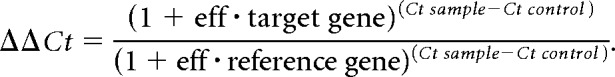
 Up to three independent reference genes were compared: L27A, which encodes a ribosomal protein of the 60S subunit (Sierra et al., 2007); OAZ-1, which encodes ornithine decarboxylase antizyme, a rate-limiting enzyme in the biosynthesis of polyamines and recently validated as reference gene in rat and human (Kwon et al., 2009); and HPRT, which encodes hypoxanthine guanine phosphoribosyl transferase (van de Moosdijk and van Amerongen, 2016). The expression of L27A, OAZ-1, and HPRT remained constant independently of time and treatments, validating their use as reference genes. In all experiments, the pattern of mRNA expression was similar using the assigned couple of reference genes, and in each experiment the reference gene that rendered lower intragroup variability was used for statistical analysis.

**Table 1.** qPCR primer sequences

	Gene	Gene Bank	Amplicon size	Sequence 5′-3′
Reference genes	OAZ1	NM_008753	51	Fwd AGCGAGAGTTCTAGGGTTGCC
				Rev CCCCGGACCCAGGTTACTAC
	L27A	BC086939	101	Fwd TGTTGGAGGTGCCTGTGTTCT
				Rev CATGCAGACAAGGAAGGATGC
	HPRT	NM_013556.2	150	Fwd ACAGGCCAGACTTTGTTGGA
				Rev ACTTGCGCTCATCTTAGGCT
Phagocytosis receptors	P2Y12	NM_027571	88	Fwd GCAGAACCAGGACCATGGAT
				Rev CTGACGCACAGGGTGCTG
	MerTK	NM_008587.1	131	Fwd AAGGTCCCCGTCTGTCCTAA
				Rev GCGGGGAGGGGATTACTTTG
	Axl	NM_009465.4	86	Fwd GTTGGTGTCTGGAGGATGGG
				Rev TGTGTGTCCTTATGGGCTGC
Peptides and hormones	VGF	NM_001039385.1	74	Fwd CACCGGCTGTCTCTGGC
				Rev AAGGAAGCAGAAGAGGACGG
	Cartpt	NM_013732.7	106	Fwd GCGCTATGTTGCAGATCGAAG
				Rev GCGTCACACATGGGGACTTG
	FGF2	NM_008006.2	113	Fwd CGGCTGCTGGCTTCTAAGTG
				Rev AGTGCCACATACCAACTGGAG
Trophic factors	VEGFA	NM_001025250.3	88	Fwd GGCCTCCGAAACCATGAACT
				Rev CTGGGACCACTTGGCATGG
	PDGFα	NM_008808.3	94	Fwd TACCCCGGGAGTTGATCGAG
				Rev TCAGCCCCTACGGAGTCTATC
	IGF-1	NM_010512.4	122	Fwd TTACTTCAACAAGCCCACAGG
		NM_184052.3		Rev GTGGGGCACAGTACATCTCC
		NM_001111274.1		
		NM_001111275.1		
		NM_001111276.1		
	EGF	NM_010113.3	136	Fwd GGACTGAGTTGCCCTGACTC
				Rev CAATATGCATGCACACGCCA
	GDNF	NM_010275.2	145	Fwd CGCTGACCAGTGACTCCAA
				Rev TGCCGATTCCTCTCTCTTCG
Matrix protein	Mmp3	NM_010809.2	88	Fwd ACCCAGTCTACAAGTCCTCCA
				Rev GGAGTTCCATAGAGGGACTGA
Surface ligands	Jag1	NM_013822.5	119	Fwd TTCAGGGCGATCTTGCATCA
				Rev CACACCAGACCTTGGAGCAG
	Dll4	NM_019454.3	113	Fwd GGTTACACAGTGAGAAGCCAGA
				Rev GGCAATCACACACTCGTTCC
Cytokines	Csf3	NM_009971.1	70	Fwd GCAGCCCAGATCACCCAGAAT
				Rev TGCAGGGCCATTAGCTTCAT
	IL1β	NM_000576.2	72	Fwd AGATGAAGTGCTCCTTCCAGG
				Rev GGTCGGAGATTCGTAGCTGG
	IL6	NM_000600.3	107	Fwd GAAAGCAGCAAAGAGGCACTG
				Rev TTCACCAGGCAAGTCTCCTCAT
	TNFα	NM_000594.3	142	Fwd TGCACTTTGGAGTGATCGGC
				Rev GCTTGAGGGTTTGCTACAACA
	TGFβ	NM_000660.5	112	Fwd TCCTGGCGATACCTCAGCAA
				Rev CAATTTCCCCTCCACGGCTC

List of primers used to amplify reference genes, phagocytosis receptors, peptides and hormones, trophic factors, matrix protein, surface ligands, and cytokines. The gene name, Gene Bank accession number, amplicon size, and sequence are listed.

##### Immunofluorescence.

Six series of 50-μm-thick coronal sections of mouse brains were cut using a Leica VT 1200S vibrating blade microtome (Leica Microsystems). Fluorescent immunostaining was performed following standard procedures (Sierra et al., 2010; Beccari et al., 2018). Free-floating vibratome sections were blocked in permeabilization solution (0.3% Triton X-100, 0.5% BSA in PBS; all from Sigma-Aldrich) for 3 h at room temperature (RT), and then incubated overnight with the primary antibodies diluted in the permeabilization solution at 4°C. For BrdU labeling an antigen retrieval procedure was performed by incubating in 2 m HCl for 30 min at 37°C and then washing with 0.1 m sodium tetraborate for 10 min at RT before the blockade of the sections. After overnight incubation with primary antibodies, brain sections were thoroughly washed with 0.3% Triton in PBS. Next, the sections were incubated with fluorochrome-conjugated secondary antibodies and DAPI (5 mg/ml; Sigma-Aldrich) diluted in the permeabilization solution for 3 h at RT. After washing with PBS, the sections were mounted on glass slides with Dako Cytomation Fluorescent Mounting Medium.

Primary microglial cultures were fixed for 10 min in 4% PFA and then transferred to PBS. Fluorescent immunostaining was performed following standard procedures (Abiega et al., 2016; Beccari et al., 2018). Coverslips with primary microglial cultures were blocked in 0.1% Triton X-100, 0.5% BSA in PBS for 30 min at RT. The cells were then incubated with primary antibodies in permeabilization solution (0.2% Triton X-100, 0.5% BSA in PBS) for 1 h at RT, rinsed in PBS and incubated in the secondary antibodies containing DAPI (5 mg/ml) in the permeabilization solution for 1 h at RT. After washing with PBS, primary cultures were mounted on glass slides with Dako Cytomation Fluorescent Mounting Medium.

For fluorouridine labeling, SH-SY5Y were treated with 2 mm 5′-fluorouridine (Sigma-Aldrich) for 30 min. Afterward, cells were fixed in 4% PFA with 0.5% Triton X-100. The immunofluorescence was performed as described with primary microglial cultures and anti-BrdU primary antibody was used to detect fluorouridine.

NPC cultures were fixed for 10 min in 4% PFA and then transferred to PBS. Coverslips containing the cells were blocked in blocking solution (0.5% Triton X-100, 3% BSA in PBS) for 1 h at RT, and then incubated overnight with the primary antibodies diluted in the permeabilization solution (0.2% Triton X-100, 3% BSA in PBS) at 4°C. After overnight incubation, coverslips were allowed to warm at RT and were thoroughly rinsed in PBS. Next, the coverslips were incubated with fluorochrome-conjugated secondary antibodies and DAPI (5 mg/ml; Sigma-Aldrich) diluted in the permeabilization solution for 2 h at RT. After washing with PBS, the coverslips were mounted on glass slides with Dako Cytomation Fluorescent Mounting Medium.

##### Western blot.

CM-treated NPCs were directly lysed in RIPA buffer containing protease and phosphatase inhibitor cocktail (100×; ThermoFisher Scientific). Cells were sonicated for 5 s and then centrifuged (10,000 × *g*, 10 min). Solubilized protein was quantified in triplicates by BCA Assay Kit (ThermoFisher Scientific) at 590 nm using a microplate reader (Synergy HT, BioTek). Ten to 15 μg of protein (denatured with β-mercaptoethanol) were loaded onto Tris-glycine gradient polyacrylamide gels (8–16%; ThermoFisher Scientific) and run at 120 V for 90 min. Protein samples were then blotted to nitrocellulose membranes (0.45 μm pore size; ThermoFisher Scientific) at 220 mA for 2 h. Transfer efficiency was verified by Ponceau S (Sigma-Aldrich) staining. For immunoblotting, membranes were rinsed in Tris-Buffered Saline containing 0.1% Tween 20 (TBS-T; Sigma-Aldrich) and then blocked for 1 h in TBS-T containing 5% powder milk. Membranes were afterward incubated with rabbit primary antibodies for REST (1:500; EMD, Millipore), and phosphorylated Smad 1/5/9 (1:500; Cell Signaling Technology), and mouse primary antibodies for Smad 1 (1:500; Santa Cruz Biotechnology), Ascl1 (1:500; BD Biosciences), and β-actin (1:5000; Sigma-Aldrich), in TBS-T containing 4% BSA overnight (4°C, shaker). Next day, membranes were rinsed and incubated with horseradish peroxidase-conjugated anti-rabbit (1:5000) and anti-mouse (1:5000) secondary antibodies (Cell Signaling Technology) in TBS-T containing 5% powder milk. After rinsing membranes, protein was visualized by enhanced chemiluminescence using Supersignal West Femto Maximum Sensitivity Substrate (ThermoFisher Scientific) in a ChemiDoc imaging system (Bio-Rad). Band intensity was quantified using the Gel Analyzer method of Fiji software. Phospho-Smad 1/5/9 levels were normalized to total levels of Smad 1. β-actin was used as loading control.

##### Image analysis.

All fluorescence immunostaining images were collected using an Olympus FluoView or a Leica SP8 laser-scanning microscope using a 40× oil-immersion objective and a *z*-step of 0.7 μm. All images were imported into Adobe Photoshop 7.0 in Tiff format. Brightness, contrast, and background were adjusted equally for the entire image using the “brightness and contrast” and “levels” controls from the “image/adjustment” set of options without any further modification. For tissue sections, two to three 20-μm-thick *z*-stacks of the sections containing the septal hippocampus from one vibratome series was analyzed (usually 6 slices, spanning from −1 to −2.5 mm in the AP axes, from bregma), to avoid variability due to the differential regulation of neurogenesis in the septal and temporal regions of the hippocampus. For primary cultures, over 4–5 *z*-stacks were obtained per coverslip.

##### Phagocytosis analysis *in vivo* and *in vitro*.

The analysis of phagocytosis *in vivo* was performed as described in a series containing the six most septal sections (Abiega et al., 2016; Beccari et al., 2018). Apoptotic cells were defined based on their nuclear morphology after DAPI staining as cells in which the chromatin structure (euchromatin and heterochromatin) was lost and appeared condensed and/or fragmented (pyknosis/karyorrhexis). Phagocytosis was defined as the formation of an enclosed, three-dimensional pouch of microglial processes surrounding an apoptotic cell. In tissue sections, the number of apoptotic cells, phagocytosed cells, BrdU+ cells, and microglia were estimated using unbiased stereology in the volume of the DG contained in the *z*-stack (determined by multiplying the thickness of the stack by the area of the DG at the center of the stack using ImageJ, Fiji). To obtain the absolute numbers, this density value was then multiplied by the volume of the septal hippocampus (spanning from −1 to −2.5 mm in the AP axes, from bregma; ∼6 slices in each of the 6 series), which was calculated using Fiji from a Zeiss Axiovert epifluorescent microscope images collected at 20×. *In vitro*, the percentage of phagocytic microglia was defined as cells with pouches containing apoptotic SH-SY5Y nuclei and/or CM-DiI particles (Beccari et al., 2018).

##### Neurogenesis analysis *in vivo* and *in vitro*.

The analysis of neurogenesis *in vivo* was performed as described previously (Encinas and Enikolopov, 2008; Abiega et al., 2016; Beccari et al., 2018). Six sections from one series containing the septal hippocampus were analyzed in all experiments except in mice injected with microglia CM, in which only the three tissue sections closest to the injection site were analyzed. Proliferation was assessed by BrdU^+^ cell quantification; neural stem cells were identified by the expression of the markers Nestin and glial fibrillary acidic protein (GFAP) and their radial morphology for cell quantification; neuroblast were assessed by doublecortin (DCX)^+^ cell quantification and morphology to classify them in AB, CD, or EF neuroblasts (Plümpe et al., 2006); neurons were assessed by NeuN^+^ cell quantification. The proliferation of either of these populations was assessed by their mentioned staining combined with BrdU. Numbers of cells were estimated using unbiased stereology in the volume of the DG of the *z*-stack, which was determined by multiplying the thickness of the *z*-stack (12 μm) by the area of the DG at the center of the stack using the software ImageJ (Fiji). *In vitro*, the effect of microglia-derived conditioned media on neuroprogenitor cells was analyzed considering both their morphology and the expression of cell-specific markers. Percentages of the different morphologies present in the population were obtained as well as the percentages of the different cell markers (nestin, GFAP, DCX, S100β) per morphology.

##### Statistical analysis.

SigmaPlot (Systat Software) was used for statistical analysis. Data were tested for normality and homoscedasticity. When the data did not comply with these assumptions, a logarithmic transformation was performed and the data were analyzed using parametric tests. In the case of IL-6 mRNA expression, normality was not achieved with the logarithmic transformation and the data were analyzed using a Kruskal–Wallis ranks test, followed by Tukey test as a *post hoc*. In the rest of the cases, two-sample experiments were analyzed by Student's *t* test and more than two-sample experiments by ANOVA. In two-way and three-way ANOVA, when interactions between factors were found, the analysis of the relevant variable was split into several one-way ANOVAs and Holm–Sidak method was used as a *post hoc*. The transformation used (if any), the test used, the comparison performed, the value of the statistical and the *p* values are shown in Tables 2–5 and 7–9. Only *p* < 0.05 is reported to be significant. Data are shown as mean ± SEM.

**Table 2.** Statistics for Figures 1 and 2

Figure	Parameter	Groups	Statistical test	Statistic	*p*
1*B*	Ph index	WT vs P2Y12 KO (1 m + 1 d)	Unpaired *t* test	*t*_(6)_ = 13.5	*p* < 0.001
	Ph capacity		Unpaired *t* test	*t*_(6)_ = 5.08	*p* = 0.002
	Apoptosis		Unpaired *t* test	*t*_(6)_ = 1.17	*p* = 0.287
	Microglia		Unpaired *t* test	*t*_(6)_ = −0.05	*p* = 0.964
1*C*	Ph index	WT vs MerTK/Axl KO (1 m)	Unpaired *t* test	*t*_(4)_ = −3.97	*p* < 0.001
	Ph capacity		Unpaired *t* test	*t*_(4)_ = 2.90	*p* = 0.044
	Apoptosis		Unpaired *t* test	*t*_(4)_ = −3.97	*p* = 0.017
	Microglia		Unpaired *t* test	*t*_(4)_ = 0.98	*p* = 0.382
1*E*	Neuroblasts	WT vs P2Y12 KO (1 m + 1 d)	Unpaired *t* test	*t*_(6)_ = 10.83	*p* < 0.001
	Proliferating Neuroblasts		Unpaired *t* test	*t*_(6)_ = 3.51	*p* = 0.013
1*F*	Neuroblasts	WT vs MerTK/Axl KO (1 m)	Unpaired *t* test	*t*_(4)_ = 2.97	*p* = 0.041
	Proliferating Neuroblasts		Unpaired *t* test	*t*_(4)_ = 0.38	*p* = 0.723
2*B*	Newborn cells	WT vs P2Y12 KO (1 m + 4 w)	Unpaired *t* test	*t*_(8)_ = 2.72	*p* = 0.026
	Newborn neurons		Unpaired *t* test	*t*_(8)_ = 2.68	*p* = 0.028
2*C*	Ph index	WT vs P2Y12 KO (1 m + 4 w)	Unpaired *t* test	*t*_(6)_ = 7.45	*p* < 0.001
	Ph capacity		Unpaired *t* test	*t*_(6)_ = 9.93	*p* < 0.001
	Apoptosis		Unpaired *t* test	*t*_(6)_ = 1.17	*p* = 0.287
	Microglia		Unpaired *t* test	*t*_(6)_ = −0.05	*p* = 0.964
2*E*	Ph index	WT vs P2Y12 KO (7 m)	Unpaired *t* test	*t*_(8)_ = 4.21	*p* = 0.003
	Apoptosis		Unpaired *t* test	*t*_(8)_ = −0.18	*p* = 0.863
	Microglia		Unpaired *t* test	*t*_(8)_ = −0.95	*p* = 0.375
2*G*	Neuroblasts		Unpaired *t* test	*t*_(8)_ = 3.14	*p* = 0.014
2*I*	P2Y12	GFP+ vs GFP−	Unpaired *t* test	*t*_(4)_ = 552,3	*p* < 0.001
	MerTK		Unpaired *t* test	*t*_(4)_ = 14.91	*p* < 0.001
	Axl		Unpaired *t* test	*t*_(4)_ = 1.82	*p* = 0.144

**Table 3.** Statistics for Figure 3

Figure	Parameter	Groups	Statistical test	Statistic	*p* value
3*E*	Ph index	Control vs iKO (1 m + 1 d)	Unpaired *t* test	*t*_(4)_ = 18.40	*p* < 0.001
	Ph capacity		Unpaired *t* test	*t*_(4)_ = 3.40	*p* = 0.027
	Apoptosis		Unpaired *t* test	*t*_(4)_ = −2.17	*p* = 0.096
	Microglia		Unpaired *t* test	*t*_(4)_ = 1.17	*p* = 0.308
3*F*	Ph index	Control vs iKO (1 m + 4 w)	Unpaired *t* test	*t*_(5)_ = 19.56	*p* < 0.001
	Ph capacity		Unpaired *t* test	*t*_(5)_ = 4.53	*p* = 0.006
	Apoptosis		Unpaired *t* test	*t*_(5)_ = −2.40	*p* = 0.062
	Microglia		Unpaired *t* test	*t*_(5)_ = 1.33	*p* = 0.240
3*H*	Proliferating cells	Control vs iKO (1 m + 1 d)	Unpaired *t* test	*t*_(4)_ = −3.79	*p* = 0.019
	Proliferating neuroblasts		Unpaired *t* test	*t*_(4)_ = −3.30	*p* = 0.030
3*I*	Neuroblasts	Control vs iKO (1 m + 1 d)	Unpaired *t* test	*t*_(4)_ = −0.54	*p* = 0.616
3*K*	Newborn cells	Control vs iKO (1 m + 4 w)	Unpaired *t* test	*t*_(8)_ = 1.77	*p* = 0.114
	Newborn neurons		Unpaired *t* test	*t*_(8)_ = 0.63	*p* = 0.547
3*L*	BrdU^+^ yield	Control vs iKO (1 m + 4 w)	Unpaired *t* test	*t*_(8)_ = 5.95	*p* < 0.001

**Table 4.** Statistics for Figure 8

Figure	Parameter	Groups	Statistical test	Statistic	*p*
8*C*	VGF (log10)	C vs Ph3 h vs Ph24 h	One-way ANOVA	*F*_(2,9)_ = 527.4	*p* < 0.001
	Cartpt (log10)		One-way ANOVA	*F*_(2,8)_ = 19.37	*p* < 0.001
	FGF2		One-way ANOVA	*F*_(2,9)_ = 5.55	*p* = 0.027
	VEGF (log10)		One-way ANOVA	*F*_(2,9)_ = 89.23	*p* < 0.001
	PDGFa (log10)		One-way ANOVA	*F*_(2,9)_ = 5.57	*p* = 0.027
	IGF1 (log10)		One-way ANOVA	*F*_(2,9)_ = 153.2	*p* < 0.001
	EGF (log10)		One-way ANOVA	*F*_(2,9)_ = 4.99	*p* = 0.035
	GDNF (log10)		One-way ANOVA	*F*_(2,9)_ = 128.8	*p* < 0.001
	Mmp3 (log10)		One-way ANOVA	*F*_(2,9)_ = 147.9	*p* < 0.001
	Jag1		One-way ANOVA	*F*_(2,9)_ = 15.26	*p* = 0.001
	Csf3 (log10)		One-way ANOVA	*F*_(2,9)_ = 242.7	*p* < 0.001
	IL-1β (log10)		One-way ANOVA	*F*_(2,9)_ = 86.82	*p* < 0.001
	IL-6		Kruskal–Wallis	*H*_(2)_ = 0.20	*p* = 0.011
	TNFα (log10)		One-way ANOVA	*F*_(2,9)_ = 25.50	*p* < 0.001
	TFGβ (log10)		One-way ANOVA	*F*_(2,9)_ = 29.04	*p* < 0.001

**Table 5.** Statistics for Figure 9

Figure	Parameter	Groups	Statistical test	Statistic	*p*
9*C*	Proliferation	MicroC vs MicroPh vs DMEM	One-way ANOVA	*F*_(2,6)_ = 14.94	*p* = 0.005
9*D*	Proliferation	Treatment × cell types	Two-way ANOVA	*F*_treat × cell(4,18)_ = 16.99	*p* < 0.001
				*F*_treat(2,18)_ = 0.00	*p* = 1
				*F*_cell(2,18)_ = 34.60	*p* < 0.001
	Proliferation MicroC	GFAP only vs nestin^+^ vs unlabelled	One-way ANOVA	*F*_(2,6)_ = 8.43	*p* = 0.018
	Proliferation MicroPH			*F*_(2,6)_ = 35.90	*p* < 0.001
	Proliferation MicroDMEM			*F*_(2,6)_ = 46.23	*p* < 0.001
9*F*	Differentiation	Treatment × cell types × time	Three-way ANOVA	*F*_treat × cell × time(6,48)_ = 0.245	*p* = 0.959
				*F*_treat × cell(6,48)_ = 138.69	*p* < 0.001
				*F*_treat × time(2,48)_ = 0.14	*p* = 0.870
				*F*_cell × time(2,48)_ = 1.26	*p* = 0.300
				*F*_treat(2,48)_ = 4.77	*p* = 0.013
				*F*_cell(3,48)_ = 194.79	*p* < 0.001
				*F*_time(1,48)_ = 0.44	*p* = 0.510
	Bipolar	MicroC vs MicroPh vs DMEM	One-way ANOVA	3 d *F*_(2,8)_ = 336.60	*p* < 0.001
				5 d *F*_(2,8)_ = 70.58	*p* < 0.001
	Stellate		One-way ANOVA	3 d *F*_(2,8)_ = 35.29	*p* < 0.001
				5 d *F*_(2,8)_ = 29.17	*p* < 0.001
	Early ramified		One-way ANOVA	3 d *F*_(2,8)_ = 3.06	*p* = 0.121
				5 d *F*_(2,8)_ = 0.86	*p* = 0.471
	Late ramified		One-way ANOVA	3 d *F*_(2,8)_ = 2.18	*p* = 0.194
				5 d *F*_(2,8)_ = 1.54	*p* = 0.288
9*H*	Dead and live cells	Treatment × life × time	Three-way ANOVA	*F*_treat × life × time(2,24)_ = 0.589	*p* = 0.563
				*F*_treat × life(2,24)_ = 37.22	*p* < 0.001
				*F*_treat × time(2,24)_ = 4.54	*p* = 0.021
				*F*_life × time(1,24)_ = 1.92	*p* = 0.179
				F_treat(2,24)_ = 20.24	*p* < 0.001
				*F*_life(1,24)_ = 128.33	*p* < 0.001
				*F*_time(1,24)_ = 0.15	*p* = 0.702
			One-way ANOVA	3 d *F*_(2,8)_ = 8.93	*p* = 0.016
	Live	MicroC vs MicroPh vs DMEM		5 d *F*_(2,8)_ = 24.86	*p* = 0.001
				3 d *F*_(2,8)_ = 16.85	*p* = 0.003
	Dead		One-way ANOVA	5 d *F*_(2,8)_ = 1.54	*p* = 0.306

**Table 6.** Functional analysis of the phagocytic microglia transcriptome reveals changes in apoptosis

Gene symbol	FC Ph3h	FC Ph24 h	Location	Effect on apoptosis
PRDX1	1,1	2	Autologous	Anti-apoptotic
SIRT1	2	1,6	Autologous	Anti-apoptotic
SMO	1	3,6	Autologous	Anti-apoptotic
SOD2	1,2	2	Autologous	Anti-apoptotic
SPHK1	4,4	1,6	Autologous	Anti-apoptotic
UBE2B	1,4	1,5	Autologous	Anti-apoptotic
XRCC5	2,1	3,8	Autologous	Anti-apoptotic
PRNP	1,8	1,3	Auto/Hetero	Anti-apoptotic
TGM2	5,5	4,5	Auto/Hetero	Anti-apoptotic
CNTF	1,5	2,6	Heterologous	Anti-apoptotic
FGF2	4,2	3,7	Heterologous	Anti-apoptotic
FGF8	1,7	1,6	Heterologous	Anti-apoptotic
VEGFA	4,5	1,8	Heterologous	Anti-apoptotic
RARG	1,3	3	Autologous	Pleiotropic
IL6	18,7	15,8	Heterologous	Pleiotropic
BAD	1,8	2,2	Autologous	Proapoptotic
FAS	1,5	1,5	Autologous	Proapoptotic
FOXO3	21	38	Autologous	Proapoptotic
GAS1	2,7	3	Autologous	Proapoptotic
NLRP3	12	19	Autologous	Proapoptotic
PPP2CB	1,5	1,6	Autologous	Proapoptotic
PTEN	1,1	1,6	Autologous	Proapoptotic
RHOA	1	1,7	Autologous	Proapoptotic
SCRIB	1,9	2,4	Autologous	Proapoptotic
STK3	1	1,5	Autologous	Proapoptotic
TFPT	2,4	3	Autologous	Proapoptotic
GAL	1,2	1,8	Heterologous	Proapoptotic
IL1β	8,9	30,7	Heterologous	Proapoptotic

Classification of the genes related to cell death obtained from DAVID analysis. The genes were classified according to their FC, to the effects on microglia (autologous), or on the surrounding cells (heterologous) and to the positive or negative effect on apoptosis.

**Table 7.** Statistics for Figure 10

10*F*	Dead and live cells (log10)	Treatment × life × time	Three-way ANOVA	*F*_treat × life × time(4,18)_ = 0.595	*p* = 0.671
				*F*_treat × life(4,18)_ = 4.656	*p* < 0.009
				*F*_treat × time(2,18)_ = 0.265	*p* = 0.770
				*F*_life × time(2,18)_ = 1.481	*p* = 0.254
				*F*_treat(2,18)_ = 0.544	*p* = 0.590
				*F*_life(2,18)_ = 50.494	*p* < 0.001
				*F*_time(1,18)_ = 0.695	*p* = 0.415
	Live	MicroC vs MicroPh vs DMEM	One-way ANOVA	5 d (log10) *F*_(2,6)_ = 8.774	*p* = 0.017
				9 d *F*_(2,6)_ = 14.103	*p* = 0.005
	Dead		One-way ANOVA	5 d *F*_(2,6)_ = 1.476	*p* = 0.301
				9 d *F*_(2,6)_ = 8.563	*p* = 0.017
10*G*	Bipolar	MicroC vs MicroPh vs DMEM	Kruskal Wallis	5 d *H*_(2)_ = 7.624	*p* = 0.071
				9 d *H*_(2)_ = 7.624	*p* = 0.071
	Stellate		Kruskal Wallis	5 d *H*_(2)_ = 5.956	*p* = 0.025
	One-way ANOVA			9 d *F*_(2,6)_ = 15.331	*p* = 0.004
	Early ramified		One-way ANOVA	5 d *F*_(2,6)_ = 1.469	*p* = 0.303
	Kruskal Wallis			9 d *H*_(2)_ = 2.000	*p* = 0.829
				5 d *F*_(2,6)_ = 7.249	*p* = 0.025
	Late ramified		One-way ANOVA	9 d *F*_(2,6)_ = 1.880	*p* = 0.232

**Table 8.** Statistics for Figures 11–13

Figure	Parameter	Groups	Statistical test	Statistic	*p* value
11*F*	REST	MicroC vs MicroPh vs DMEM	One-way ANOVA	*F*_(3,11)_ = 0.688	*p* = 0.594
	Ascl		One-way ANOVA	*F*_(3,11)_ = 3.53	*p* = 0.068
	pSMAD/SMAD		One-way ANOVA	*F*_(3,11)_ = 27.30	*p* < 0.001
12*C*	Differentiation	Treatment × cell types × time	Three-way ANOVA	*F*_treat × cell × time(2,24)_ = 0.24	*p* = 0.787
				*F*_treat × cell (2,24)_ = 0.46	*p* = 0.636
				*F*_treat × time (2,24)_ = 0.156	*p* = 0.856
				*F*_cell × time(1,24)_ = 1.052	*p* = 0.315
				*F*_treat(2,24)_ = 6.42	*p* = 0.006
				*F*_cell(1,24)_ = 1235.66	*p* < 0.001
				*F*_time(1,24)_ = 0.361	*p* = 0.554
12*D*	CSF3 (log10)	MicroC vs MicroPH vs LPS vs MicroPH + LPS	One-way ANOVA	*F*_(3,12)_ = 44.56	*p* < 0.001
	IL-1β (log10)		One-way ANOVA	*F*_(3,12)_ = 19.44	*p* < 0.001
	IL-6		Kruskal–Wallis	*H*_(3)_ = 11.43	*p* = 0.010
	TNFα (log10)		One-way ANOVA	*F*_(3,12)_ = 52.539	*p* < 0.001
	TGFβ (log10)		One-way ANOVA	*F*_(3,12)_ = 3.57	*p* = 0.047
13*B*	Dead and live cells	Treatment × life × time	Three-way ANOVA	*F*_treat × life × time(2,24)_ = 0.512	*p* = 0.606
				*F*_treat × life(2,24)_ = 4.79	*p* = 0.018
				*F*_treat × time(2,24)_ = 0.14	*p* = 0.872
				*F*_life × time(1,24)_ = 0.90	*p* = 0.757
				*F*_treat(2,24)_ = 1.33	*p* = 0.283
				*F*_life(1,24)_ = 309.10	*p* < 0.001
				*F*_time(1,24)_ = 0.01	*p* = 0.912

**Table 9.** Statistics for Figure 14

Figure	Parameter	Groups	Statistical test	Statistic	*p*
14*C*	BrdU_2 h_ (log10)	MicroC vs MicroPh	Unpaired *t* test	*t*_(18)_ = −0.003	*p* = 0.998
14*D*	Apoptosis	MicroC vs MicroPh	Unpaired *t* test	*t*_(12)_ = −0.867	*p* = 0.403
14*F*	Stem cells	MicroC vs MicroPh	Unpaired *t* test	*t*_(18)_ = 0.42	*p* = 0.677
14*G*	Stem cell proliferation	MicroC vs MicroPh	Unpaired *t* test	*t*_(18)_ = 1.97	*p* = 0.065
14*I*	Neuroblasts (total)	MicroC vs MicroPh	Unpaired *t* test	*t*_(12)_ = 0.02	*p* = 0.984
	AB		Unpaired *t* test	*t*_(12)_ = −0.18	*p* = 0.862
	CD		Unpaired *t* test	*t*_(12)_ = −0.42	*p* = 0.679
	EF		Unpaired *t* test	*t*_(12)_ = 0.293	*p* = 0.774
14*J*	BrdU neuroblasts (log10)	MicroC vs MicroPh	Unpaired *t* test	*t*_(12)_ = −0.47	*p* = 0.650
14*M*	BrdU_4w_ (log10)	MicroC vs MicroPh	Unpaired *t* test	*t*_(17)_ = 2.41	*p* = 0.031
14*N*	Apoptosis	MicroC vs MicroPh	Unpaired *t* test	*t*_(11)_ = 0.12	*p* = 0.908
14*P*	Neuroblasts	Treatment × distance	Two-way ANOVA_rep_	*F*_treat × dist(1,11)_ = 0.20	*p* = 0.664
				*F*_dist(1,11)_ = 0.852	*p* = 0.376
				*F*_treat(1,11)_ = 14.19	*p* = 0.003
14*R*	Neuroblasts (total)	MicroC vs MicroPh	Unpaired *t* test	*t*_(18)_ = 3.38	*p* = 0.003
	AB		Unpaired *t* test	*t*_(18)_ = 1.80	*p* = 0.099
	CD		Unpaired *t* test	*t*_(18)_ = 2.09	*p* = 0.035
	EF		Unpaired *t* test	*t*_(18)_ = 4.62	*p* < 0.001
14*S*	New neurons	MicroC vs MicroPh	Unpaired *t* test	*t*_(17)_ = 2.28	*p* = 0.036

##### Statistical analysis of gene expression arrays.

To analyze the differential expression between naive (*t* = 0) and phagocytic (*t* = 3 h and *t* = 24 h) microglia groups over time the statistical analysis the maSigPro package of R/Bioconductor was used (R v3.0.3, Bioconductor release v2.13, maSigPro v1.34.1; Conesa et al., 2006). This method is based on a general regression approximation for the modeling and adjustment of the parameters required according to the type of analysis. The parameter “Time” is considered as a continuous variable, and creates a regression model of the gene response. The analysis was performed in three steps. First, genes that exhibited changes in expression over time were selected based on a *p*-corrected Benjamini–Hochberg (FDR) value. Next, for each of the genes that presented a significant change in their expression over time a regression was applied to determine their model (*R*^2^ > 0.7) to identify patterns or models of change based on time variables, obtaining 20,800 probes. Finally, the probes were selected according to their fit to the regression model. Next, the number of genes with a very high differential pattern was reduced by applying a more restrictive criterion (*R*^2^ > 0.9), obtaining 13,146. The *R*^2^ > 0.7 list was used for the identification of neurogenesis related genes and the *R*^2^ > 0.9 list was used for the study of transcriptional profile of phagocytic microglia.

##### DAVID.

The database for annotation, visualization, and integrated discovery (DAVID; https://david.ncifcrf.gov/) v6.8 provides a comprehensive set of functional annotation tools to understand biological meaning behind large list of genes. DAVID was used to generate a gene-GO term enrichment analysis that identified enriched biological themes and to highlight the most relevant GO terms associated with the array gene list. The array gene list of *R*^2^ > 0.9 was used for this gene profile analysis. The analysis of each expression pattern was performed separately and only terms with an adjusted *p* value (Benjamini–Hochberg) > 0.05 were considered significant.

##### ClueGO.

ClueGO was used to generate protein pathways and to constitute the network of pathways based on the Gene Ontology and KEGG database (Bindea et al., 2013). ClueGO is a plugin of Cytoscape (http://www.cytoscape.org/) that visualizes the non-redundant biological terms for large clusters of genes in a functionally grouped network. A ClueGO network is created with κ statistics and reflects the relationships between the terms based on the similarity of their associated genes (Mlecnik et al., 2018). Gene ontology (GO) analysis of mouse array data were performed with ClueGO v1.4 (Bindea et al., 2013) using the following parameters: enrichment/depletion two-sided hypergeometric statistical test; correction method: Benjamini–Hochberg; GO term range levels: 3–8; minimal number of genes for term selection: 10; minimal percentage of genes for term selection: 10%; κ-score threshold: 0.8; general term selection method: smallest *p* value; group method: κ; minimal number of subgroups included in a group: 3; minimal percentage of shared genes between subgroups: 50%.

## Results

### Chronic impairment of microglial phagocytosis reduces adult hippocampal neurogenesis

**Figure 1.**
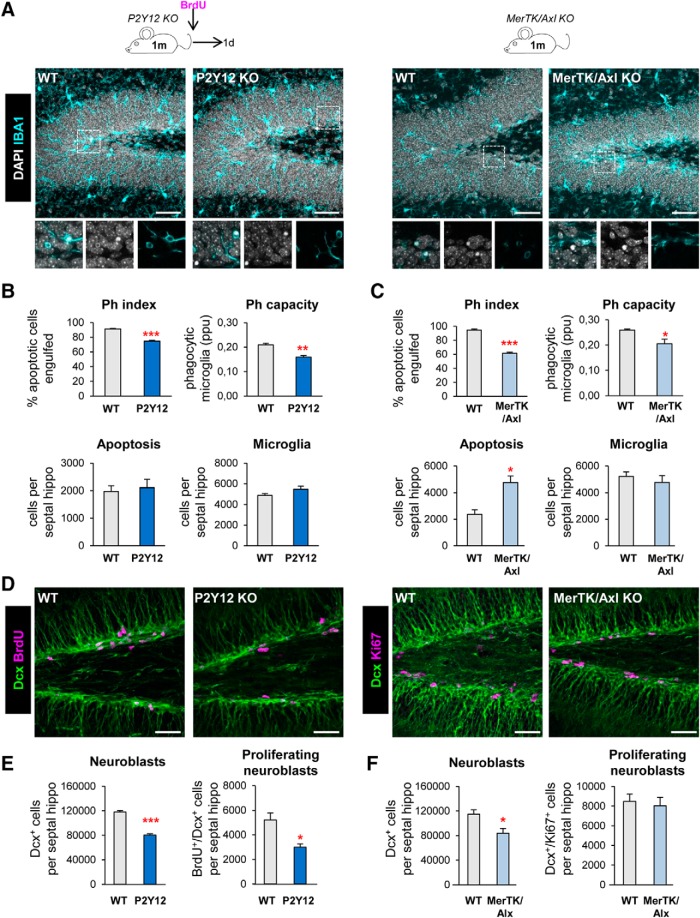
Chronic microglial phagocytosis impairment reduces adult hippocampal neurogenesis. ***A***, Representative maximum projection of confocal *z*-stack of P2Y12 and MerTK/Axl KO mice immunofluorescence in the mouse hippocampal DG at 1 month (1 m). Microglia were labeled with Iba1 (cyan) and apoptotic nuclei were detected by pyknosis/karyorrhexis (white, DAPI). ***B***, ***C***, Percentage of apoptotic cells engulfed (Ph index), weighted average of the percentage of microglia with phagocytic pouches (Ph capacity), apoptotic cells and microglia per septal hippocampus in P2Y12 KO mice (***B***) and MerTK/Axl KO mice (***C***). ***D***, Representative confocal *z*-stack of P2Y12 and MerTK/Axl KO mice immunofluorescence in the mouse hippocampal DG at 1 m. Neuroblasts were labeled with DCX (green) and proliferation was labeled with either BrdU (150 mg/kg, 24 h) or Ki67 (magenta). ***E***, Neuroblast and neuroblast proliferation in 1-month-old P2Y12 KO mice. ***F***, Neuroblast and neuroblast proliferation in 1-month-old MerTK/Axl KO mice. Scale bars: ***A***, ***D***, 50 μm (inserts, 10 μm); ***A***, left, *z* = 20 μm; ***A***, right, *z* = 17 μm; ***D***, left, *z* = 7 μm; ***D***, right, *z* = 10 μm. *N* = 3–4 mice (***B***, ***C***, ***E***, ***F***). Error bars represent mean ± SEM. *p < 0.05, **p < 0.01, ***p < 0.001 by Student's *t* test. Only significant effects are shown. Values of statistics used are shown in Table 2.

To examine the impact of microglial phagocytosis on adult hippocampal neurogenesis *in vivo*, we focused on two signaling pathways involved on phagocytosis: P2Y12 (purinergic receptor type Y12), which mediates chemotaxis toward the “find-me” signal ADP (Haynes et al., 2006); and the TAM family tyrosine kinases MerTK and Axl, which bind to phosphatidylserine adapter/bridging molecules: growth arrest specific factor 6 and protein S (Elliott et al., 2009; Fourgeaud et al., 2016). We used two transgenic mouse models in which these proteins are constitutively knocked out (P2Y12 KO and MerTK/Axl KO). We decided to study the impact of phagocytosis in young mice (1 month old), as neurogenesis and apoptosis of newborn cells and subsequent phagocytosis by microglia rapidly declines with age (Sierra et al., 2010; Beccari et al., 2017). Apoptotic cells were defined as pyknotic/karyorrhectic nuclei labeled with the DNA dye DAPI, which we have previously characterized to express other apoptosis markers such as activated caspase 3 and fractin (Sierra et al., 2010). First, we assessed phagocytosis in the hippocampus of the two KO models by quantifying the Ph index (the percentage of apoptotic cells engulfed by microglia), which is ∼90% in physiological conditions (Abiega et al., 2016), and found significantly lower Ph index in the two KO models (74.8 ± 0.9% for P2Y12, 61.5 ± 1.6% for MerTK/Axl; Fig. 1*A–C*). In addition, the microglial Ph capacity (weighted average of the number of pouches containing apoptotic cells per microglia, i.e., the average number of phagocytic pouches per microglia) was significantly reduced in both KO models (Fig. 1*A–C*). Nonetheless, we found no changes in the number of microglia and the phagocytosis reduction was small, possibly because of compensatory mechanisms resulting from the chronic depletion, and we only detected the expected increase of apoptotic cells in MerTK/Axl KO mice (Fig. 1*A–C*), possibly indicating not a complete dysfunction but a slowdown of phagocytosis.

**Figure 2.**
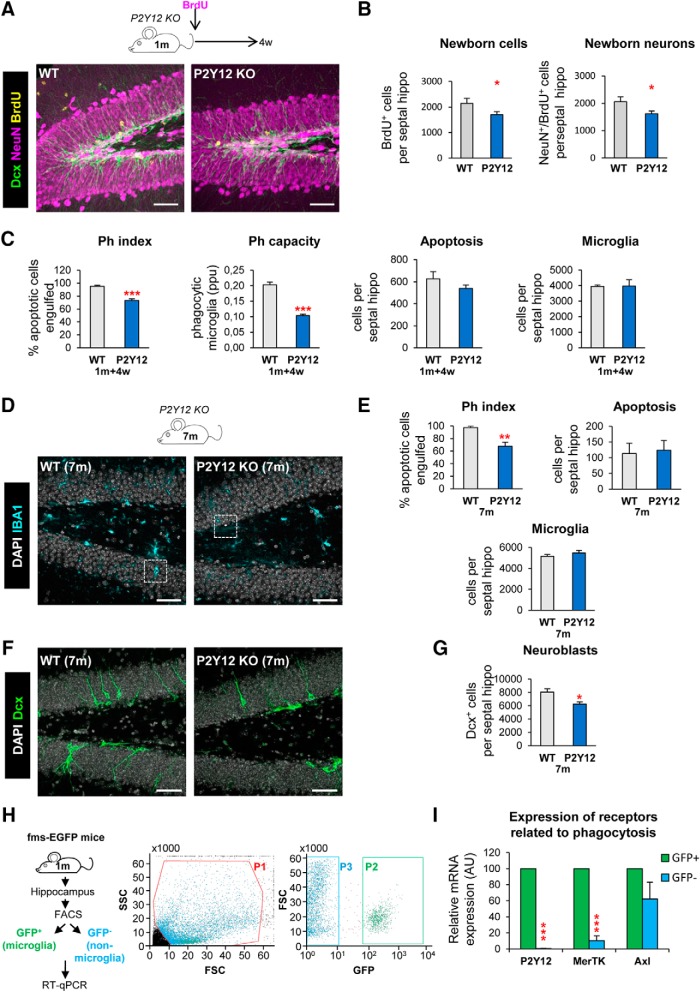
Chronic microglial phagocytosis impairment reduces adult hippocampal neurogenesis in the long term. ***A***, Representative confocal *z*-stack of P2Y12 KO mice immunofluorescence in the mouse hippocampal DG at 2 months. Neuroblasts were labeled with DCX (green), neurons were labeled with NeuN (magenta) and proliferation was labeled with BrdU (yellow; 4 weeks after BrdU injection). ***B***, New cells and new neurons (NeuN^+^, BrdU^+^) in 2-month-old P2Y12 KO mice, 4 weeks after the BrdU injection. ***C***, Percentage of apoptotic cells engulfed (Ph index), weighted average of the percentage of microglia with phagocytic pouches (Ph capacity), apoptotic cells and microglia per septal hippocampus in P2Y12 KO mice 4 weeks after BrdU injection (2m). ***D***, Representative maximum projection of confocal *z*-stack of P2Y12 KO mice immunofluorescence in the mouse hippocampal DG at 7 months (7 m). Microglia were labeled with Iba1 (cyan) and apoptotic nuclei were detected by pyknosis/karyorrhexis (white, DAPI). ***E***, Percentage of apoptotic cells engulfed (Ph index), number of apoptotic cells and microglia per septal hippocampus in 7-month-old P2Y12 KO mice. ***F***, Representative confocal *z*-stack of P2Y12 KO mice immunofluorescence in the mouse hippocampal DG at 7 months. Neuroblasts were labeled with DCX (green). ***G***, Number of neuroblasts per septal hippocampus in 7-month-old P2Y12 KO mice. ***H***, Experimental design used to isolate microglia (GFP^+^) versus non-microglial cells (GFP^−^) from 1-month-old fms-EGFP mice using flow cytometry. First, debris was excluded using the P1 gate in FSC versus SSC (left). Next, gates for GFP^+^ microglia cells (P2) and GFP^−^ non-microglial cells (P3) were defined based on the distribution of the fms-EGFP^+^ cells in EGFP versus FSC (right). ***I***, Expression of P2Y12, MerTK, and Axl in microglia (GFP^+^) versus non-microglial cells (GFP^−^) by real-time qPCR in FACS-sorted cells from fms-EGFP mice hippocampi. OAZ1 (ornithine decarboxylase antizyme 1) was selected as a reference gene. Scale bars: ***A***, ***D***, ***F***, 50 μm; ***A***, *z* = 20 μm; ***D***, ***F***, *z* = 17.5 μm. *N* = 5 mice (***A***). *N* = 4–6 mice (***E***, ***G***), *N* = 3 independent experiments (***H***; each from 8 pooled hippocampi), *p < 0.05, **p < 0.01, ***p < 0.001 by Student's *t* test. Values of statistics used are shown in Table 2.

Next, we examined hippocampal neurogenesis in these phagocytosis impaired KO models and observed that the two showed a significant decrease in the population of neuroblasts and immature neurons, labeled with DCX, compared to wild-type (WT) controls. In addition, we assessed proliferation by quantifying the number of dividing cells using either BrdU, an analog of thymidine that gets incorporated into the DNA during S phase of dividing cells (mice were injected with 150 mg/kg and killed 24 h later); or the proliferation marker Ki67^+^ (Scholzen and Gerdes, 2000). P2Y12 KO mice had a decrease in both neuroblasts (reduction of 31.7 ± 2.7%) and neuroblast proliferation (reduction of 39.3 ± 9.8%), and MerTK/Axl KO mice showed a reduction in neuroblasts (reduction of 26.2 ± 9.4%) compared to WT mice (Fig. 1*D–F*). We further studied the formation of newborn neurons using BrdU pulse-and-chase in the most robust model, the P2Y12 KO. Four weeks after the BrdU injection, when the mice were 2 months old, both total BrdU^+^ cells and newborn neurons (NeuN^+^/BrdU^+^) were reduced in P2Y12 KO mice compared to WT mice (reduction of 24.6 ± 9.7%; Fig. 2*A*,*B*), in parallel to a decrease in phagocytosis (Ph index and Ph capacity) and no changes in apoptosis nor microglia (Fig. 2*C*). The defect in neurogenesis was maintained later in life, because at 7 months, P2Y12 KO mice still showed a significant reduction in phagocytosis (Ph index) without alterations in apoptosis nor microglia (Fig. 2*D*,*E*), in parallel to a reduction in neuroblasts compared to WT mice (Fig. 2*F*,*G*).

To confirm the specificity of these results, we analyzed the expression of P2Y12, MerTK, and Axl in FACS-sorted cells from 1-month-old fms-EGFP mice, in which microglia is labeled with EGFP. We found that, P2Y12 and MerTK, but not Axl, were highly expressed in microglia compared with other cells of the hippocampal parenchyma (Fig. 2*H*,*I*), suggesting that the disruption of neurogenesis in the KO models might be attributable to the lack of these receptors in microglia. These receptors regulate multiple features of microglial physiology (Elliott et al., 2009; Fourgeaud et al., 2016). In addition, constitutive MerTK/Axl KO mice show autoimmune diseases (Rothlin and Lemke, 2010), as both MerTK and Axl are highly expressed in peripheral macrophages (http://rstats.immgen.org/Skyline/skyline.html). In addition, in the brain Axl is also expressed in astrocytes (http://www.brainrnaseq.org/). Nonetheless, P2Y12 is largely specific to microglia (http://www.brainrnaseq.org/) and P2Y12 KO mice show an impairment of microglial phagocytosis and concomitant decrease in neurogenesis up to 7 months. Thus, the similar phagocytosis impairment and neurogenesis reduction in the two KO models suggests that an intact microglial phagocytosis is necessary for the long-term maintenance of hippocampal neurogenesis.

### Acute microglial phagocytosis impairment transiently increases adult hippocampal neurogenesis

**Figure 3.**
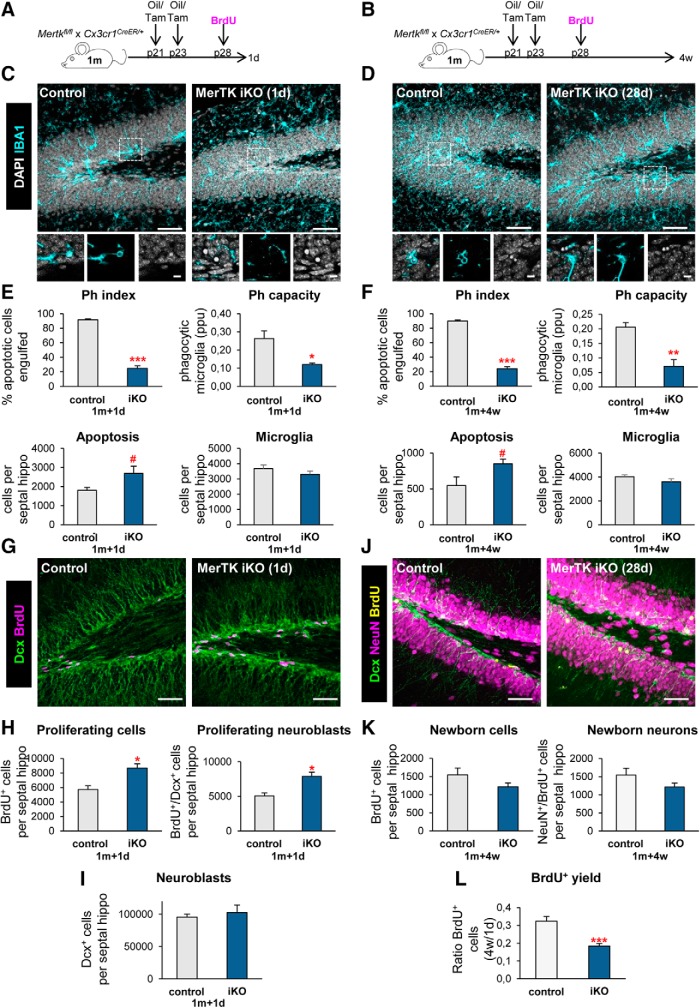
Acute microglial phagocytosis impairment transiently increases adult hippocampal neurogenesis. ***A***, ***B***, Experimental design in microglial-specific MerTK inducible KO mice, generated by crossing *Mertk^fl/fl^* and *Cx3cr1^CreER^* mice treated with tamoxifen (iKO) or corn oil (control) at P21 and P23 and injected with BrdU at p28 (100 mg/kg). Mice were killed at 1 d (***A***) or 4 weeks (***B***) after the BrdU injection. ***C***, ***D***, Representative maximum projection of confocal *z*-stack of MerTK iKO mice immunofluorescence in the mouse hippocampal DG at 1 d (***C***) and 4 weeks (***D***). Microglia were labeled with Iba1 (cyan) and apoptotic nuclei were detected by pyknosis/karyorrhexis (white, DAPI). ***E***, ***F***, Percentage of apoptotic cells engulfed (Ph index), weighted average of the percentage of microglia with phagocytic pouches (Ph capacity), apoptotic cells and microglia per septal hippocampus in MerTK iKO mice at 1 d (***E***) and 4 weeks (***F***). ***G***, Representative confocal *z*-stack of MerTK iKO mice immunofluorescence in the mouse hippocampal DG at 1 d. Neuroblasts were labeled with DCX (green) and proliferation was detected with BrdU (magenta). ***H***, Newborn cells (BrdU^+^) and newborn neuroblasts (DCX^+^, BrdU^+^) in MerTK iKO mice at 1 d post-BrdU. ***I***, Neuroblasts (DCX^+^) in MerTK iKO mice at 1 d post-BrdU. ***J***, Representative confocal *z*-stack of MerTK iKO mice immunofluorescence in the mouse hippocampal DG at 4 weeks post-BrdU. Neuroblasts were labeled with DCX (green), neurons were labeled with NeuN (magenta) and proliferation was labeled with BrdU (yellow). ***K***, Newborn cells (BrdU^+^) and newborn neurons (NeuN^+^, BrdU^+^) in MerTK iKO mice at 4 weeks post-BrdU. ***L***, BrdU+ yield was calculated as a ratio of the BrdU^+^ cells at 4 weeks over the average BrdU^+^ cells of each group at 1 d after injection. Scale bars: ***C***, ***D***, ***G***, ***J***, 50 μm (insets, 10 μm); ***C***, ***D***, *z* = 16.1 μm; ***G***, *z* = 7 mm; ***J***, *z* = 23.1 μm. *N* = 3 mice (***E***, ***H***, ***I***), *N* = 4–6 mice (***F***, ***K***, ***L***). Error bars represent mean ± SEM. #*p* = 0.096 (***E***), #*p* = 0.062 (***F***), *p < 0.05, **p < 0.01, ***p < 0.001 by Student's *t* test. Only significant effects are shown. Values of statistics used are shown in Table 3.

We then studied the effect of acute phagocytosis blockage on neurogenesis using an inducible MerTK KO model (generated by crossing *Mertk^fl/fl^* to *Cx3cr1*^*CreER*/+^ mice; Fourgeaud et al., 2016). Mice received tamoxifen (two 75 mg/kg i.p. injections) or vehicle (corn oil) at P21 and P23 to induce microglial-specific Cre-mediated depletion of *Mertk*, and one injection of BrdU at P28 to label dividing cells. Phagocytosis and neurogenesis were analyzed at 1 d and 4 weeks after BrdU administration (i.e., when mice were 1 and 2 months old, respectively; Fig. 3*A*,*B*). Phagocytosis was strongly reduced in the inducible KO mice injected with tamoxifen (iKO) compared to control mice injected with vehicle, as shown by the Ph index (73.0 ± 3.7% and 72.9 ± 2.6% reduction at 1 d and 4 weeks, respectively) and the Ph capacity (54.5 ± 3.1% and 65.6 ± 11.0% reduction at 1 d and 4 weeks, respectively), together with an accumulation of apoptotic cells (Fig. 3*C–F*). iKO mice showed no significant changes in the number of microglia but a trend toward increased apoptotic cells compared to control mice (Fig. 3*E*,*F*). Concomitantly, 1 d after the BrdU injection the number of proliferating BrdU^+^ cells and the number of DCX^+^, BrdU^+^, proliferating neuroblasts increased (51.8 ± 10.2% and 55.4 ± 12.2% increase, respectively), whereas there was no change in the total number of neuroblasts in MerTK iKO compared to control mice injected with oil (Fig. 3*G–I*). However, at 4 weeks after BrdU injection there were no significant changes in the total number of newborn cells nor newborn neurons (BrdU^+^, NeuN^+^; Fig. 3*J*,*K*). Thus, the excess of BrdU cells formed at 1 d were lost at 4 weeks, and indeed the net yield of newborn cells, calculated as a ratio of the cells at 4 weeks over the cells at 1 d, was significantly lower in iKO compared to control mice (43.1 ± 4.5% reduction; Fig. 3*L*).

This transient increase in neurogenesis in the MerTK iKO model (Fig. 3) is in apparent disagreement with the reduction of neurogenesis in the constitutive P2Y12 and MerTK/Axl KO models (Figs. 1, 2). It is important to note that in the iKO model, phagocytosis is acutely and strongly impaired in the adult hippocampus, whereas in the constitutive KO models the effect on phagocytosis is low, chronic and from embryonic development. In addition, other differences could explain the contrast between the models, including targeting peripheral immune cells in MerTK/Axl KO mice, but not in the P2Y12 KO mice or the MerTK iKO mice; or the fact that iKO mice were generated in a heterozygous CX3CR1 background, which reduces basal neurogenesis (Rogers et al., 2011). Importantly, the administration of tamoxifen, used to activate the Cre recombinase, does not affect neurogenesis (Rotheneichner et al., 2017). Nonetheless, the data from the three models altogether suggests that microglial phagocytosis participates in the regulation of adult hippocampal neurogenesis.

### Phagocytosis of apoptotic cells triggers the expression of neurogenic modulatory factors by microglia *in vitro*

**Figure 4.**
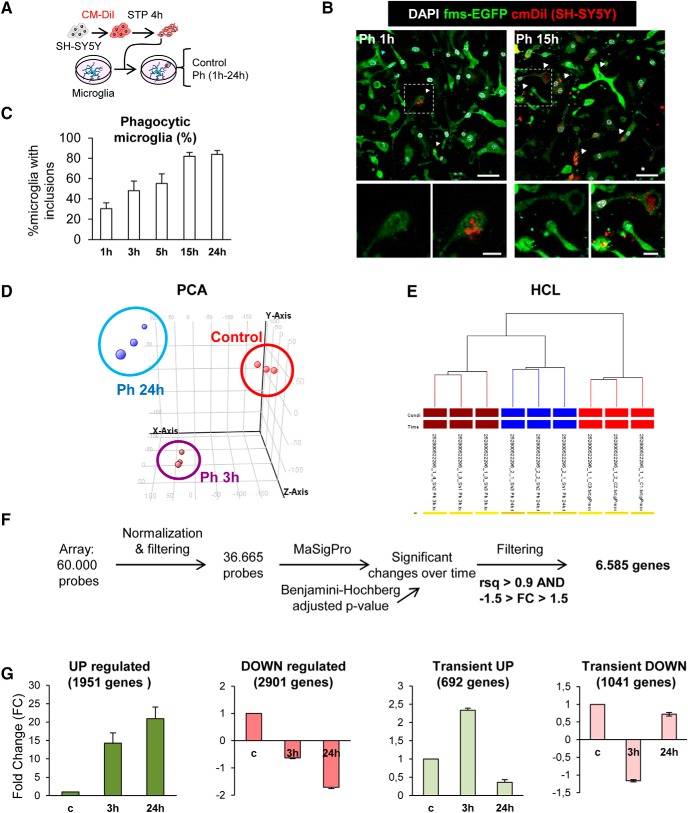
Phagocytosis assay with a human neural cell line (SH-SY5Y). ***A***, Experimental design of the phagocytosis assay. ***B***, Representative confocal microscopy images of primary microglia (GFP; green) fed with SH-SY5Y, which were previously labeled with CM-DiI (red) and treated with STP (4 h, 3μM) for the induction of apoptosis (pyknosis/karyorrhexis; DAPI, white). Arrowheads, phagocytosed apoptotic SH-SY5Y cells. ***C***, Percentage of microglia with CM-DiI and/or DAPI inclusions along a time course. Only fully closed pouches with particles within were identified as phagocytosis. ***D***, PCA of the different replica of the samples: Control microglia, Ph3h, and Ph24h. ***E***, Hierarchical clustering (HCL) of the different replica of the samples control microglia (blue), Ph3h (brown), and Ph24h (red). ***F***, Representation of the strategy followed to screen genes from the gene array. ***G***, FC mean of the genes classified under the UP, DOWN, transient UP, and transient DOWN regulation patterns. Scale bars: ***B***, 30 μm (inserts, 10 μm). *N* = 3 independent experiments (***C***–***G***).

To study the mechanism by which microglial phagocytosis regulates neurogenesis, we developed a xenogenic *in vitro* model of phagocytosis of apoptotic cells (Beccari et al., 2018), in which mouse primary microglia were fed for different lengths of time (1–24 h) with a human neuronal line (SH-SY5Y), previously labeled with CM-DiI and treated with STP (4 h, 3 μm) to induce apoptosis (Fig. 4*A*,*B*). In this model, pooled telencephalic microglia were used in the cultures, disregarding possible specific effects of hippocampal microglia. When fed with apoptotic human cells, microglia were phagocytic as early as 1 h (30.2 ± 5.8%), a percentage that kept increasing until 24 h (83.8 ± 3.6%; Fig. 4*C*). We then performed a mouse-specific genome-wide transcriptomic analysis using gene expression mouse-specific arrays to compare naive versus phagocytic microglia.

Hence, we compared the genome-wide transcriptome naive versus phagocytic microglia (3 and 24 h) using gene expression arrays. Hierarchical clustering and principal component analysis (PCA) of the transcriptome of control, Ph3h and Ph24h microglia showed strong differences in the clustering of the expression profile of three groups (Fig. 4*D*,*E*). To analyze which particular genes were different among the three experimental groups, we searched for array probes with significant changes over time using a *p*-corrected Benjamini–Hochberg value and a polynomial regression model to identify time patterns (Conesa et al., 2006). We obtained 10,000 significantly regulated probes with a restrictive criterion of *R*^2^ > 0.9 and a fold-change (FC) > 1.5 or <−1.5, eventually obtaining 6585 genes that presented significant changes over time (Fig. 4*F*).

We classified these genes according to four main expression patterns (Fig. 4*G*): UP (upregulation both at 3 and 24 h), DOWN (downregulation in both time points), transient UP (upregulation at 3 h and downregulation at 24 h), and transient DOWN (downregulation at 3 h and upregulation at 24 h). Genes in the UP expression pattern showed the largest average FC changes among all patterns, some of them even reaching 8000 FC at 24 h. The rest of the patterns had on average more modest changes.

**Figure 5.**
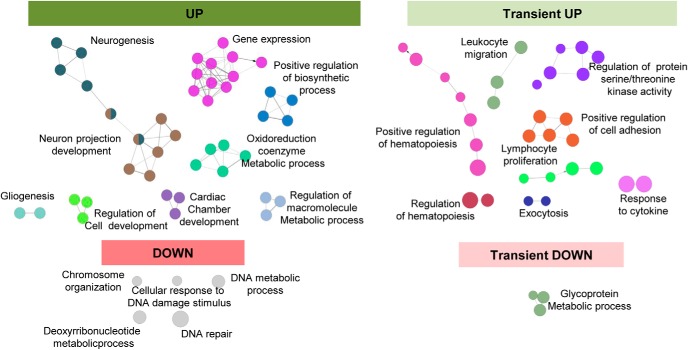
Functional analysis of phagocytic microglia using ClueGO. Charts show the interactions among the significantly different functions for the four main expression patterns. Biological functions are visualized as colored nodes linked to related groups based on their κ score level. The node size reflects the enrichment significance of the term and functionally related groups are linked. Non-grouped terms are shown in gray.

**Figure 6.**
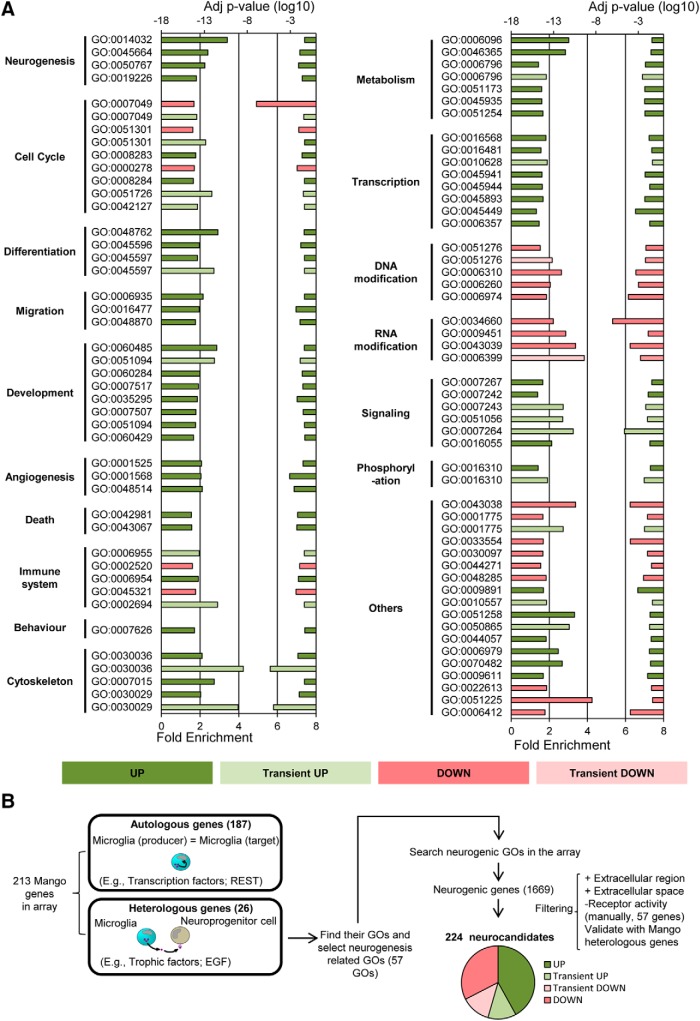
Functional analysis of phagocytic microglia using DAVID and MANGO. ***A***, Functional analysis of phagocytic microglia using DAVID software. Left axis represents the fold enrichment of each biological function and right axis represents the adjusted *p* value of each GO term. Key for the GO terms: GO:0014032, neural crest cell development; GO:0045664, regulation of neuron differentiation; GO:0050767, regulation of neurogenesis; GO:0019226, transmission of nerve impulse; GO:0007049, cell cycle; GO:0051301, cell division; GO:0008283, cell proliferation; GO:0000278, mitotic cell cycle; GO:0008284, positive regulation of cell proliferation; GO:0051726, regulation of cell cycle; GO:0042127, regulation of cell proliferation; GO:0048762, mesenchymal cell differentiation; GO:0045596, negative regulation of cell differentiation; GO:0045597, positive regulation of cell differentiation; GO:0006935, chemotaxis; GO:0016477, cell migration; GO:0048870, cell motility; GO:0060485, mesenchyme development; GO:0051094, positive regulation of developmental process; GO:0060284, regulation of cell development; GO:0007517, muscle organ development; GO:0035295, tube development; GO:0007507, heart development; GO:0051094, positive regulation of developmental process; GO:0060429, epithelium development; GO:0001525, angiogenesis; GO:0001568, blood vessel development; GO:0048514, blood vessel morphogenesis; GO:0042981, regulation of apoptosis; GO:0043067, regulation of programmed cell death; GO:0006955, immune response; GO:0002520, immune system development; GO:0006954, inflammatory response; GO:0045321, leukocyte activation; GO:0002694, regulation of leukocyte activation; GO:0007626, locomotory behavior; GO:0030036, actin cytoskeleton organization; GO:0007015, actin filament organization; GO:0030029, actin filament-based process; GO:0006096, glycolysis; GO:0046365, monosaccharide catabolic process; GO:0006796, phosphate metabolic process; GO:0006796, phosphate metabolic process; GO:0051173, positive regulation of nitrogen compound metabolic process; GO:0045935, positive regulation of nucleobase, nucleoside, nucleotide and nucleic acid metabolic process; GO:0051254, positive regulation of RNA metabolic process; GO:0016568, chromatin modification; GO:0016481, negative regulation of transcription; GO:0010628, positive regulation of gene expression; GO:0045941, positive regulation of transcription; GO:0045944, positive regulation of transcription from RNA polymerase II promoter; GO:0045893, positive regulation of transcription, DNA-dependent; GO:0045449, regulation of transcription; GO:0006357, regulation of transcription from RNA polymerase II promoter; GO:0051276, chromosome organization; GO:0006310, DNA recombination; GO:0006260, DNA replication; GO:0006974, response to DNA damage stimulus; GO:0034660, ncRNA metabolic process; GO:0009451, RNA modification; GO:0043039, tRNA aminoacylation; GO:0006399 tRNA metabolic process; GO:0007267, cell–cell signaling; GO:0007242, intracellular signaling cascade; GO:0007243, protein kinase cascade; GO:0051056, regulation of small GTPase-mediated signal transduction; GO:0007264, small GTPase-mediated signal transduction; GO:0016055, Wnt receptor signaling pathway; GO:0016310, phosphorylation; GO:0043038, amino acid activation; GO:0001775, cell activation; GO:0033554, cellular response to stress; GO:0030097, hemopoiesis; GO:0044271, nitrogen compound biosynthetic process; GO:0048285, organelle fission; GO:0009891, positive regulation of biosynthetic process; GO:0010557, positive regulation of macromolecule biosynthetic process; GO:0051258, protein polymerization; GO:0050865, regulation of cell activation; GO:0044057, regulation of system process; GO:0006979, response to oxidative stress; GO:0070482, response to oxygen levels; GO:0009611, response to wounding; GO:0022613, ribonucleoprotein complex biogenesis; GO:0051225, spindle assembly; GO:0006412, translation. Left axis represents the fold enrichment of each biological function and right axis represents the adjusted *p* value of each GO term. Only statistically significant changes are shown. ***B***, Diagram depicting the strategy followed to search for potential modulators of neurogenesis produced by phagocytic microglia in the arrays. The filtering started by differentiating the heterologous and autologous genes in the MANGO database. Then, GO terms related to neurogenesis were selected for the heterologous MANGO genes. Afterward, the molecules that presented the neurogenic GO terms were searched in the array. Finally, the candidate genes were filtered only to select those that appeared extracellularly (heterologous genes), and genes with receptor activity were manually discarded.

Next, we performed a functional analysis of the phagocytic microglia transcriptome using the ClueGO network (Fig. 5) and DAVID (Fig. 6*A*). These analyses revealed a number of functional biological pathways associated with each of the four main expression patterns of phagocytic microglia including downregulation in pathways related to DNA and chromosomes and upregulation of different functions associated with metabolism and chromatin remodeling. Interestingly, different studies suggesting metabolic changes in phagocytes upon the uptake of apoptotic cells have recently emerged (Morioka et al., 2018). Importantly, for many upregulated genes, ClueGO revealed specific terms like “generation of neurons”, “neuron differentiation” or “neuron development” that were grouped under the term “neurogenesis” and had a direct interrelation with “neuron projection development” group.

We then focused on the identity of the neurogenesis-related genes using the following strategy (Fig. 6*B*). To identify phagocytosis-related potential regulators of neurogenesis, we used MANGO (The Mammalian Adult Neurogenesis Gene Ontology), a database of 259 genes already described to be involved in the regulation of adult hippocampal neurogenesis (Overall et al., 2012). Of the MANGO genes, 213 were found significantly regulated in our arrays. We then filtered those MANGO genes by disregarding those encoding for autologous proteins (i.e., acting on the same cell, such as transcription factors) and focusing on those encoding heterologous proteins (i.e., acting on neighbor cells, such as secreted molecules). We first applied this criterion to MANGO, and found 26 heterologous genes that had been previously identified to regulate adult hippocampal neurogenesis. To further extend the list of heterologous genes outside MANGO that could be potential regulators of neurogenesis, we looked into the filtered gene array list (*R*^2^ > 0.9 and −1.5 > FC > 1.5), searched the GO terms associated with each MANGO heterologous gene, and selected those terms that could be related to different steps of the neurogenic process (proliferation, differentiation, migration, chemotaxis, survival, and development). MANGO heterologous genes encompassed 57 different neurogenesis-related GO terms, such as growth factor activity (GO:0008083), nervous system development (GO:0007399), learning (GO:0007612), memory (GO:0007613), cell proliferation (GO:0008283), cell differentiation (GO:0030154), and neuron development (GO:0048666).

**Figure 7.**
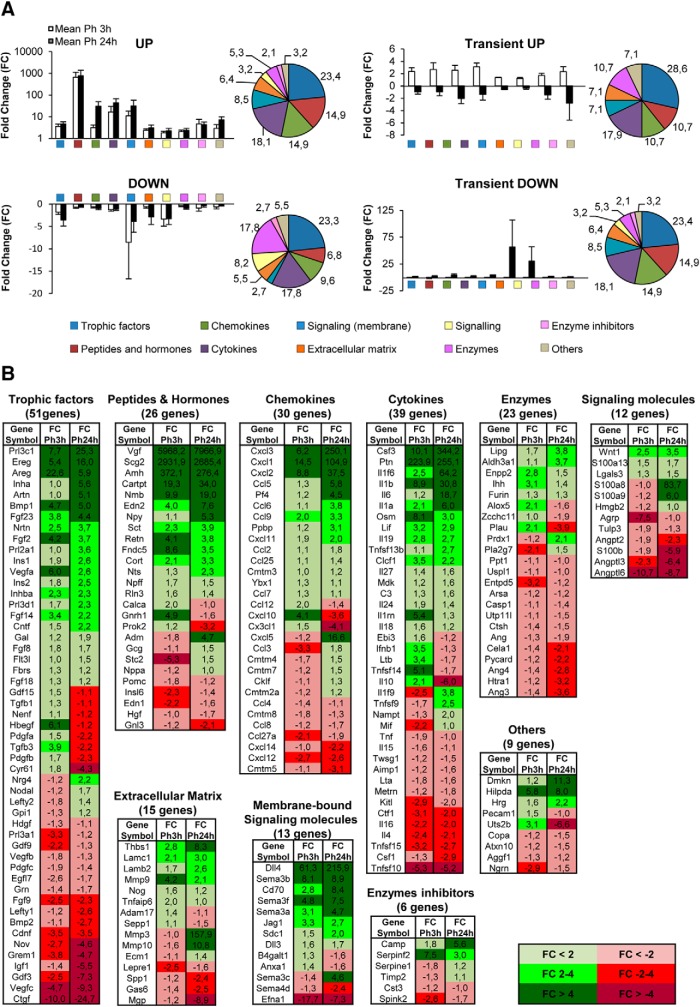
Phagocytosis-related candidates include trophic factors and peptides and hormones. ***A***, Classification of the 224 potential modulators of neurogenesis. “Trophic factor” was the category with the highest percentage of genes in every regulatory pattern, however, the category “Peptides and hormones” included genes with the highest FC changes in the UP regulation pattern. ***B***, The 224 candidates classified by their identity and FC.

We then searched for heterologous genes belonging to each of these 57 categories in our arrays, using a less-restrictive list of 20,800 probes (*R*^2^ > 0.7, no screening of FC). We finally obtained 224 genes with differential expression between naive and phagocytic microglia, which were heterologous and whose function had been previously involved in neurogenesis (based on the GO terms). The 224 candidate genes were classified according to their main regulatory expression patterns: 94 UP, 73 DOWN, 28 transient-UP, and 29 transient-DOWN genes (Fig. 6*B*). In the four regulation patterns, the majority of the genes were categorized as trophic factors (between 23 and 29% in all regulatory patterns; Fig. 7*A*,*B*). We also found cytokines, chemokines, peptides, and hormones as the main gene types of the candidates. Despite the fact that trophic factors were the largest percentage in each regulation pattern, they showed a similar and rather low mean FC compared to the other categories. Only the upregulated peptides and hormones revealed a large mean of 800 FC at both 3 and 24 h of phagocytosis. These data suggest that peptides and hormones were the most likely molecules to perform modulatory functions in the neurogenic niche by phagocytic microglia.

**Figure 8.**
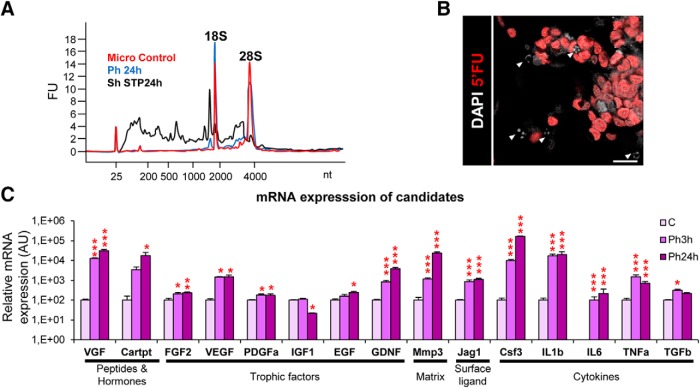
Validation of transcriptional changes induced by phagocytosis. ***A***, Electropherogram obtained by a bioanalyzer comparing the RNA profile (nt, nucleotides) of control and phagocytic microglia as well as apoptotic SH-SY5Y (treated with 3μM STP for 24 h). ***B***, Representative confocal images of FU^+^ active transcription sites of SH-SY5Y cells treated with STP (3μM, 4 h) for apoptosis induction. Nuclei were labeled with DAPI (white), cell death was detected by pyknosis/karyorrhexis (white, DAPI; arrowheads), and transcription sites were detected by FU (red). ***C***, mRNA expression levels of the candidates selected for validation by real-time qPCR. *N* = 4 independent experiments. HPRT was selected as a reference gene. Scale bar, 20 μm. *N* = 4 independent experiments (***F***). Error bars represent mean ± SEM. *p < 0.05, **p < 0.01, ***p < 0.001 by Holm–Sidak *post hoc* test of (after one-way ANOVA was significant at *p* < 0.05). Only significant effects are shown. Values of statistics used are shown in Table 4.

To disregard the possible detection of residual mRNA from the (human) apoptotic cells, we checked the sequence of the 60,000 array probes against the human transcriptome by BLAST. We found that 96% of the probes had low homology to the human transcriptome (MegaBlast homology < 5%). In addition, we analyzed the RNA integrity in apoptotic cells using a bioanalyzer, and found that whereas naive and phagocytic (24 h) microglia had the expected 18S and 28S rRNAs profile, apoptotic SH-SY5Y cells (24 h) showed a smear typical of RNA degradation (Fig. 8*A*). Finally, we analyzed whether apoptotic cells could synthesize new mRNA using 5′-fluorouridine (FU; a uridine analog that integrates at transcription sites; Fig. 8*B*). Apoptotic SH-SY5Y, unlike live cells, did not exhibit nuclear FU labeling, evidencing that they were not transcriptionally active. Altogether, the RNA profiling and analysis of transcription in apoptotic cells strongly suggests that although the gene arrays used could virtually detect up to 4% of mRNAs from human apoptotic cells, their lack of residual RNA would result solely in the detection of microglial-specific transcriptional changes after phagocytosis.

Finally, we validated the mRNA expression of the candidates in naive and phagocytic microglia by real-time qPCR. We selected a subset of genes for validation based both on their high FC in the array and/or the well known neurogenesis modulatory potential described in the literature. We found that the expression pattern of the selected candidates determined by real-time qPCR was largely in agreement with that obtained in the arrays. Among the genes with the largest mRNA expression were the neuropeptide VGF, the matrix metalloprotease 3, and the cytokine colony stimulating factor 3 (Fig. 8*C*), reinforcing the notion that phagocytosis promotes the production of neurogenic modulators by microglia.

### The secretome from phagocytic and naive microglia drives neuroprogenitor cells toward different fates *in vitro*

**Figure 9.**
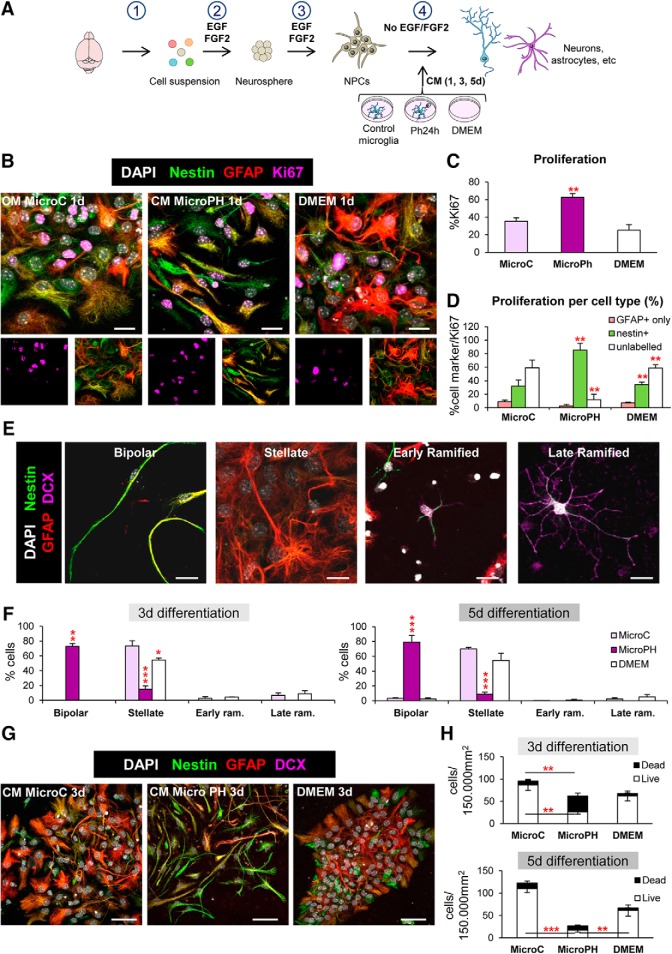
Effect of phagocytic microglia secreted factors on neurogenesis *in vitro*. ***A***, Experimental design of the *in vitro* neurogenesis assay: (1) brain disaggregation; (2) neurosphere proliferation; (3) dissociation, plating, and proliferation of NPCs for 48 h; (4) differentiation in the presence of conditioned media (CM) from control microglia (microC) or 24 h phagocytic microglia (microPh). ***B***, Representative confocal microscopy images of NPCs treated with CM microC or microPH after 1 d. DMEM was used as control. ***C***, Percentage of cells labeled with Ki67 over total cells labeled with DAPI. ***D***, Percentage of different cell markers over total Ki67 population: nestin^+^ (with or without GFAP), GFAP only, or unlabeled. ***E***, Representative confocal microscopy images of the different morphologies observed in the neurogenesis assay images. ***F***, Percentage of the different cell types found after 3 and 5 d treatment with CM microC or microPH. Early/late ram refers to early/late ramified cells. ***G***, Representative confocal microscopy images of NPCs treated with CM microC or microPH after 3 d. ***H***, Density of live and dead cells (determined by pyknosis/karyorrhexis) after CM treatment for 3 and 5 d. Scale bars: ***B***, 50 μm; ***E***, 9 μm; ***B***, *z* = 11.9 μm; ***G***, *z* = 20 μm. *N* = 3 independent experiments (***C***, ***D***, ***F***, ***H***). Error bars represent mean ± SEM. Two-way ANOVA (treatment × cell types, ***D***) and three-way ANOVA (treatment × cell types × time, ***F***; and treatment × life × time, ***H***) showed interactions between the different factors and thus the data were split into several one-way ANOVAs. *p < 0.05, **p < 0.01, ***p < 0.001 by Holm–Sidak *post hoc* test versus MicroC group (after one-way ANOVA was significant at *p* < 0.05). Values of statistics used are shown in Table 5.

Of the 224 heterologous candidates, 83.5% belonged to the secretome, suggesting an important role of the phagocytic microglial secretome on the modulation of neurogenesis. We thus directly tested the effect of the phagocytic microglial secretome on neurogenesis *in vitro*. To model neurogenesis, we used a monolayer of NPC cultures derived from disaggregated neurospheres, obtained from whole P0–P1 brains and allowed them to proliferate 48 h in DMEM/F12 with trophic factors EGF/FGF2 (Babu et al., 2011). We first performed a neurogenesis differentiation assay in which NPCs were allowed to differentiate during a time course (1–5 d) in the presence of conditioned media from control [naive microglia (CM microC)] and phagocytic [24 h phagocytic microglia (CM microPH)] microglia (Fig. 9*A*). DMEM was used as an internal control, because microglia were cultured in this media. After 1 d of differentiation, cultures in CM microPH maintained higher levels of proliferation than CM microC as observed by a higher proportion of cells labeled with the proliferation marker Ki67^+^ (Fig. 9*B*,*C*). Most of the proliferating cells in microPH cultures expressed nestin, a marker of progenitor cells, stem cells, and reactive astrocytes (Encinas and Sierra, 2012; Lopez-Atalaya et al., 2018; Fig. 9*D*). We then followed the progeny of those populations at 3 and 5 d and labeled them with cell identity markers: nestin, GFAP, a marker of astrocytes (Encinas and Sierra, 2012) and DCX, a marker of neuroblasts (Brown et al., 2003; Fig. 9*E*). We found that CM microC treatment mainly produced GFAP^high^, nestin^+/−^, stellate cells both at 3 and 5 d, as well as a small percentage of DCX ramified cells. In contrast, in CM microPH cultures the majority of the cells were nestin^high^, GFAP^+^, with a bipolar morphology (Fig. 9*F*,*G*). Cell death (apoptosis) was observed in all conditions, as has been noted before in this type of cultures upon growth factor withdrawal-induced differentiation (Babu et al., 2011). However, higher rates of apoptosis were found in NPCs cultured in CM microPH, which resulted in a lower cell density (Fig. 9*H*). Importantly, the majority (57%) of cell death-related upregulated heterologous genes were anti-apoptotic (Table 6), suggesting that the phagocytic microglia secretome did not directly induce NPCs apoptosis. Together, the above results (Fig. 9) results indicate that naive microglia led to the production of stellate cells, resembling astrocytes in culture, and a small proportion of ramified DCX^+^-expressing cells, corresponding to an immature stage of the neuronal lineage. In contrast, phagocytic microglia drove NPCs toward a unique bipolar cell type, expressing nestin and GFAP but never DCX, which is characteristic of both astrocytes and undifferentiated progenitor cells (Encinas and Sierra, 2012).

### The secretome from phagocytic microglia drives neuroprogenitor cells toward an astrocytic phenotype *in vitro*

To precisely identify bipolar cells produced by CM microPH, we performed several studies to characterize their phenotype: labeling with the mature astrocytic marker (S100β; Raponi et al., 2007), a multipotency assay, response to stimuli by calcium imaging, and Western blot analysis of neural and astrocyte-committed transcription factors.

**Figure 10.**
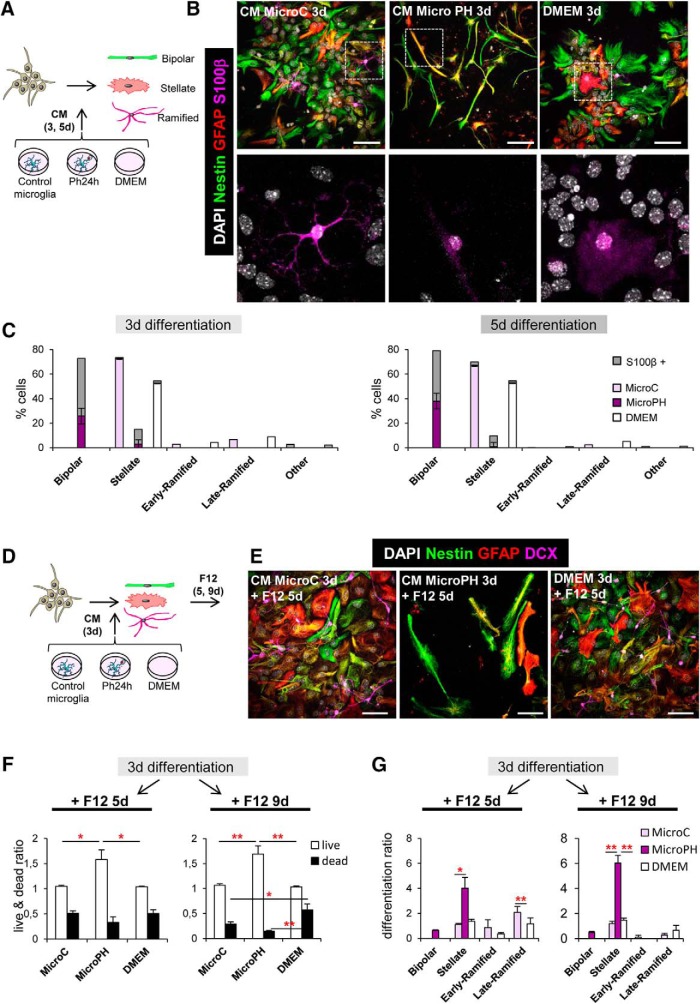
Characterization of CM cell types by S100β and multipotency assays. ***A***, Experimental design of the *in vitro* neurogenesis assay for S100β staining. ***B***, Representative confocal microscopy images of NPCs treated with CM microC or microPH. DMEM was used as control. ***C***, Percentage of expression of S100β in the different cell types found after 3 and 5 d treatment with CM microC or microPH. The category “Other” refers to cells with strong S100β expression, no GFAP and a ramified morphology suggest that they may be oligodendrocytes (Hachem et al., 2005). ***D***, Experimental design of the *in vitro* multipotency assay. ***E***, Representative confocal microscopy images of NPCs treated with CM microC, microPH or DMEM followed by 5 d of DMEM/F12. ***F***, Ratio of live/dead cell density over the cells at 3 d after each treatment. ***G***, Differentiation ratio of each phenotype after 3 d treatment with CM microC or microPH followed by 5 or 9 d DMEM/F12. Scale bars: ***B***, ***E***, 20 μm (inserts in ***B***, 10 μm); ***B***, ***F***, *z* = 9 μm. *N* = 3 independent experiments (***C***, ***F***, ***G***). Error bars represent mean ± SEM. *p < 0.05, **p < 0.01. Values of statistics used are shown in Table 7.

First, we tested whether bipolar cells could be mature astrocytes and stained the CM-treated cultures with S100β, a marker of mature astrocytes (Raponi et al., 2007; Fig. 10*A*). In CM microC few stellate cells were S100β^+^ and exhibited rather dim labeling, suggesting that they were still immature astrocytes (Fig. 10*B*,*C*). In addition, in CM microC cultures, we observed a small percentage of cells that did not express GFAP, expressed high levels of S100β and had several branched processes, that may possibly be oligodendrocytes (Hachem et al., 2005). On the other hand, in CM microPH cultures the majority of both bipolar and stellate cells had a very dim S100β staining and oligodendrocyte-like cells were not found. Therefore, the faint S100β expression in bipolar cells triggered by conditioned media from phagocytic microglia suggested that they could be immature astrocytes.

Second, we analyzed the multipotency of bipolar cells. After 3 d of differentiation into stellate, ramified, and bipolar cells in CM microC and microPH, we switched the culture media to DMEM/F12 culture media (the regular media to grow neurospheres) without trophic factors and allowed cells to differentiate for another 5–9 d (Fig. 10*D–G*). We found that after 5–9 d in DMEM/F12, cells derived from microPH CM had lower rates of cell death than cultures derived from microC and DMEM treatments, calculated as a ratio over the number of cells at 3 d in each culture (Fig. 10*F*). We then analyzed the multipotency of the cultures by calculating the ratio of change of each cell type after DMEM/F12. CM microC-derived cultures presented similar ratios of stellate cells and ramified cells after 5–9 d in DMEM/F12 (Fig. 10*G*). In contrast, cultures derived from microPH CM had a strong increase in the ratio of differentiation into stellate cells while neuroblasts were not found (Fig. 10*G*). These data strongly suggest that bipolar cells produced after treatment with the phagocytic microglia secretome were unlikely to be prototypical neuroprogenitors because they only gave rise to astrocytes but not to neuron-committed cells.

**Figure 11.**
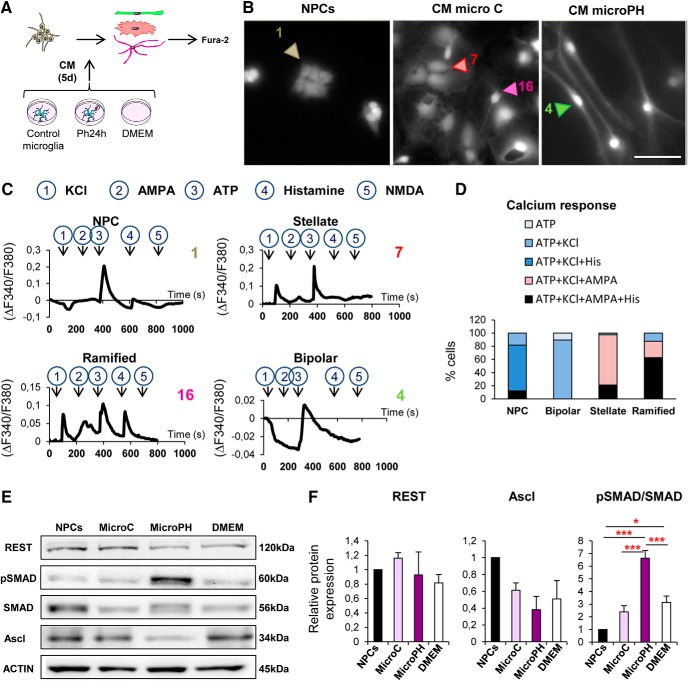
Characterization of CM cell types by calcium imaging and late survival/differentiation assays. ***A***, Experimental design of the *in vitro* calcium imaging assay. NPCs were treated with CM microC or microPH for 5 d and the resulting stellate, ramified and bipolar cells were incubated and loaded with Fura-2 AM and afterward, cells were challenged with KCl, AMPA, ATP, histamine, and NMDA to measure their Ca^+2^ response. ***B***, Representative epifluorescence microscopy images of neuroprogenitors treated with CM microC or microPH for 5 d. Freshly dissociated NPCs were used as control. ***C***, Calcium responses to consecutive stimuli (KCl, AMPA, ATP, histamine, NMDA) determined as a ratio of Fura2 fluorescence of cells shown in ***B***. ***D***, Percentage of cell phenotypes responding to each stimulus (38 stellate cells, 8 ramified cells, 19 bipolar cells, and 33 NPCs; pooled from *N* = 2 independent experiments). The baseline was calculated as the mean of the first 60 s of recording for each cell. Only peaks that increase or decrease three times the SEM of the baseline were considered as a positive response. ***E***, Representative blots showing relative levels of REST, Ascl, phospho-SMAD1/5/9, and SMAD1 in NPCs treated with CM microC or microPH for 3 d. ***F***, Quantification of the relative expression of REST, Ascl and the ratio phospho-SMAD/total-SMAD in NPCs treated with CM microC or microPH for 3 d. β-actin was used as a loading control. Scale bar, 20 μm. *N* = 2 independent experiments (***D***, pooled cells), *N* = 3 independent experiments (***F***). Error bars represent mean ± SEM. *p < 0.05, ***p < 0.001 by Holm–Sidak *post hoc* test (after one-way ANOVA was significant at *p* < 0.05). Values of statistics used are shown in Table 8.

Third, we used calcium responses to different cell-specific stimuli to characterize bipolar cells triggered by microPH medium. We used Fura-2 AM, a cell permeant calcium indicator: KCl, which triggers an intracellular Ca^+2^ response in excitable cells (De Melo Reis et al., 2011); AMPA, which depolarizes neurons expressing the corresponding glutamate receptors (Bloodgood and Sabatini, 2008); ATP, which activates purinergic receptors in astrocytes and neurons (De Melo Reis et al., 2011); histamine, which triggers intracellular Ca^+2^ response in immature cells through histamine receptor, highly expressed on immature/stem cells and embryonic stem cells (Eiriz et al., 2011); and NMDA (Fig. 11*A–D*). We examined the calcium response to these stimuli of bipolar and stellate cells, as well as freshly dissociated NPCs as control. We found that 69% of the freshly dissociated NPCs depolarized in response to ATP and histamine, and became hyperpolarized in response to KCl. The majority of stellate cells depolarized in response to KCl, AMPA, and ATP, and the majority of ramified cells responded to all stimuli except NMDA, most likely because they were still immature neuroblasts. On the other hand, in CM microPH-treated cultures, the majority of bipolar cells highly depolarized when incubated with ATP and hyperpolarized when incubated with KCl (Fig. 11*C*,*D*). These data show that bipolar cells have similar features as both NPCs and astrocytes, suggesting an intermediate phenotype.

Finally, we performed Western blot analysis of NPCs as well as CM microC and CM microPH cultures of different fate-committing transcription factors: REST (RE1 silencing transcription factor 1), a repressor of neuronal genes that is highly expressed in astrocytes (Kohyama et al., 2010); Ascl (ASC1-like protein), which is related to neuronal fate commitment (Liu et al., 2015); and SMAD1, which is highly phosphorylated in differentiating astrocytes (Kohyama et al., 2010). In the CM-treated NPC cultures, we found no differences for REST and Ascl, but the pSMAD/SMAD ratio was significantly increased in CM microPH-treated cells compared to CM microC (Fig. 11*E*,*F*), suggesting that CM microPH bipolar cells were committed to the astrocytic lineage. All together, these *in vitro* data demonstrate that at the cellular level, the microglial phagocytosis secretome promotes the inhibition of neural-committed cells and resulted in the differentiation of NPCs into astrocytes.

### Cytokines are unlikely to drive the bipolar phenotype triggered by phagocytic microglia conditioned media

**Figure 12.**
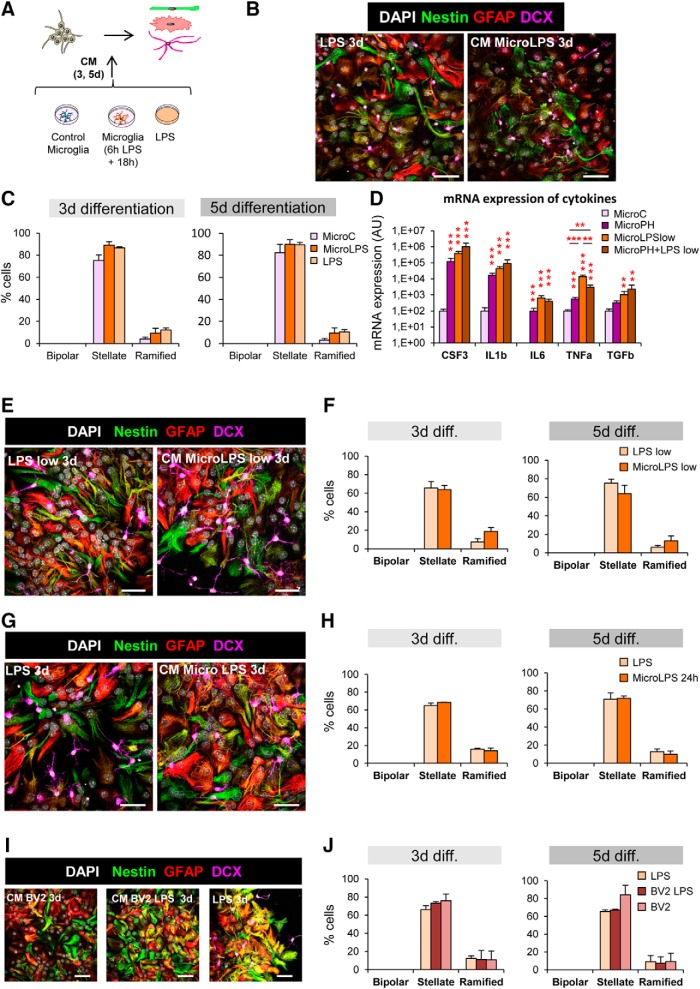
Effect of CM microLPS on neurogenesis *in vitro*. ***A***, Experimental design of the *in vitro* neurogenesis assay. ***B***, Representative confocal microscopy images of neuroprogenitors treated with CM MicroC, CM MicroLPS 6 h + 18 h (1 μg/ml) or LPS alone (1 μg/ml; 24 h). ***C***, Percentage of cell types found after 3 or 5 d treatment with CM MicroC, CM MicroLPS (6 h + 18 h), LPS. The group “bipolar cells” is included for visualization purposes, but as this cell type was not found with any of the treatments, it was not included in the statistical analysis. Three-way ANOVA (treatment × life × time) showed interactions between the different factors and thus the data were split into several one-way ANOVAs, which showed no significant effect of the treatment. ***D***, mRNA expression levels of selected cytokines by real-time qPCR in control microglia (microC), 24 h phagocytic microglia (microPH), as well as control and phagocytic microglia treated with LPS (150 ng/ml, 18 h). HPRT was selected as a reference gene. ***E***, Representative confocal microscopy images of NPCs treated with CM from LPS treated microglia or LPS alone (low concentration: 150 ng/ml; 18 h). ***F***, Quantification of the different cell types found after 3 or 5 d treatment with CM from LPS treated microglia or LPS. ***G***, Representative confocal microscopy images of NPCs treated with CM MicroLPS or LPS (1 μg/ml; 24 h). ***H***, Quantification of the different cell types found after 3 or 5 d treatment with CM MicroLPS or LPS (high concentration: 1 μg/ml; 24 h). ***I***, Representative confocal microscopy images of neuroprogenitors treated with CM BV2, CM BV2 LPS high or LPS high (1 μg/ml; 24 h). ***J***, Quantification of the different cell types found after 3 or 5 d treatment with CM BV2, CM BV2 LPS, or LPS. Scale bars: ***B***, ***E***, ***G***, ***I***, 20 μm, *z* = 6.3 μm. *N* = 3 independent experiments (***C***), *N* = 4 independent experiments (***D***), *N* = 2 independent experiments (***F***, ***H***, ***J***). Error bars represent mean ± SEM. **p < 0.01, ***p < 0.001 by Holm–Sidak *post hoc* test (after one-way ANOVA was significant at *p* < 0.05). Only significant effects are shown. Values of statistics used are shown in Table 8.

We next focused on characterizing the nature of the phagocytosis secretome. As observed in the arrays (Figs. 7, 8), phagocytic microglia expressed the mRNA of several cytokines, such as Csf3, IL-1β, IL-6, TNF-α, or TGF-β among others. In addition to phagocytosis, these molecules are also released by microglia upon inflammatory stimuli and some have already been reported to impair neurogenesis (Ekdahl et al., 2003; Monje et al., 2003). To directly compare cytokine expression induced by phagocytosis and by a classical inflammatory stimulus such as LPSs (bacterial lipopolysaccharides) we treated NPCs with the CM of primary microglia that had been pretreated with LPSs (1 μg/ml; Monje et al., 2003) for 6 h to trigger the inflammatory response, and then changed to fresh media for another 18 h to ensure that the CM would not contain any leftover LPSs (Fig. 12*A*). We found that NPC cultures treated with LPSs or CM microLPS produced a majority of stellate cells and a small proportion of ramified cells, although there were no differences between the two treatments in terms of cell-type proportion and numbers (Fig. 12*B*,*C*). CM microLPS treatments did not trigger bipolar cells, strongly suggesting that cytokines were unrelated to the effect of the phagocytosis secretome on NPCs.

Unexpectedly, neither LPSs nor CM microLPS reduced the number of neuroblasts compared to CM microC (Fig. 12*B*,*C*). These were surprising results because proinflammatory cytokines are well documented to exert detrimental consequences for neurogenesis (Ekdahl et al., 2003; Monje et al., 2003). To disregard that this discrepancy resulted from different LPS concentration or exposure time compared with prior publications, we performed a series of LPS-based experiments in which we used first, a lower LPS dosage (150 ng/ml for 18 h; Fraser et al., 2010) that produced a very similar cytokine expression as phagocytosis in microglia (Fig. 12*D–F*); second, the exact LPS dosage and time (1 μg/ml, 24 h) described by Monje et al. (2003), where they found a reduction in DCX^+^ cells after CM microLPS treatment (Fig. 12*G*,*H*); and third, the paradigm described by Monje et al. (2003), who used the BV2 cell line instead of primary microglia (Fig. 12*I*,*J*), although they used hippocampal NPC cultures derived from adult rats. None of the LPS or CM LPS treatments gave rise to bipolar cells, strongly suggesting that cytokines are highly unlikely to drive the bipolar phenotype triggered by the phagocytic microglia secretome.

### The phagocytosis secretome reduces neuronal differentiation

**Figure 13.**
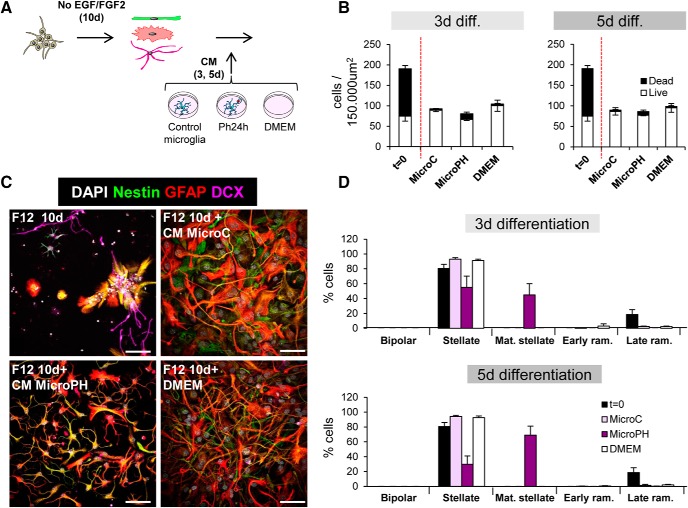
Effect of phagocytic microglia secreted factors on late neurogenesis *in vitro*. ***A***, Experimental design of the *in vitro* late survival and differentiation assay. ***B***, Density of live and dead cells found after 10 d of DMEM/F12 followed by 3–5 d of CM microC or microPH and DMEM. The number of cells before adding the CM (*t* = 0) is shown as a control. ***C***, Representative confocal microscopy images of NPCs treated for 10d with DMEM/F12 followed by 3–5 d of CM microC, microPH, or DMEM. Top, Left, DMEM/F12 treatment of 10 d, before adding any CM. ***D***, Percentage of cell types found after 10 d of DMEM/F12 followed by 3–5 d of CM microC or microPH and DMEM. The number of cells before adding the CM (*t* = 0) is shown as a control. Mat stellate designates stellate cells with mature (more branched) morphology, and early/late ram designates early/late ramified cells. Scale bars: 20 μm, *z* = 6.3 μm. *N* = 3 independent experiments (***B***, ***D***). Three-way ANOVA (treatment × life × time, ***F***) showed interactions between the different factors and thus the data were split into several one-way ANOVAs, which showed no significant effect of the treatment. Data in ***D*** could not be normalized because some cell categories were only present in particular treatments (i.e., the mature stellate phenotype only occurred in MicroPH groups). Error bars represent mean ± SEM. Values of statistics used are shown in Table 8.

We next characterized the effect of the CMs at later stages of neurogenesis using a late survival/differentiation assay in which NPCs were allowed to differentiate for 10 d into neuroblasts and astrocytes using DMEM/F12 without trophic factors. At this stage (*t* = 0), the cultures exhibited a high percentage of cell death (65.8 ± 2.4%) and the majority of the cells had a stellate morphology. These differentiated cultures were then treated for 3 and 5 d with CM microC and microPH as well as DMEM for positive control (Fig. 13*A*,*B*). Importantly, treatment with microPH did not result in higher levels of apoptosis than microC or DMEM (Fig. 13*B*). Cultures treated with CM microC presented a vast majority of stellate cells and few ramified cells. In contrast, CM microPH-treated cultures showed no DCX^+^ cells, a small percentage of stellate cells and a majority of stellate cells with a more mature morphology (more complex ramifications; Fig. 13*C*,*D*). Nonetheless, the gene array data did not support a direct induction of astrogenesis. The functions “gliogenesis” and “glial cell differentiation” were significantly upregulated in the ClueGo analysis (Fig. 5), but the majority of the genes found under those categories were autologous, and therefore, their overexpression would only modulate the microglial cells expressing them. As the astrocytic lineage is the default differentiation mode of neural stem cells (Bonaguidi et al., 2011; Encinas et al., 2011), these data suggest that the phagocytosis secretome inhibited neuronal differentiation, indirectly promoting astrocyte differentiation.

### Neurogenic modulatory factors secreted by phagocytic microglia *in vitro* alter neurogenesis *in vivo*

**Figure 14.**
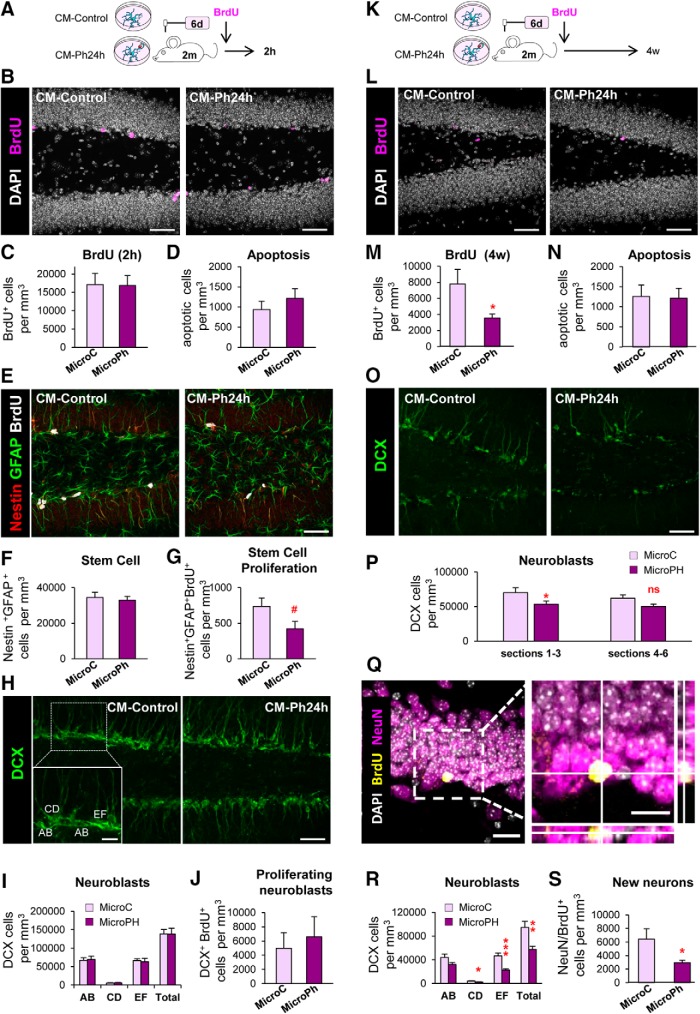
Acute and long-term effects of phagocytic microglia secreted molecules on neurogenesis *in vivo*. ***A***, Experimental design used for the administration of CM microC or microPH by osmotic pumps to 2-month-old fms-EGFP mice. ***B***, Representative confocal images of cell proliferation after the CM treatments for 6 d. Cell nuclei were labeled with DAPI (white) and BrdU was used as a proliferative marker (magenta). ***C***, BrdU^+^ cell density after CM microC or microPH treatment. ***D***, Apoptotic cell density after CM microC or microPH treatment. ***E***, Representative confocal images of stem cells labeled with nestin (red) and GFAP (green). ***F***, Stem cell density after CM microC or microPH treatment. ***G***, Proliferating stem cell (nestin^+^, GFAP^+^, BrdU^+^) density after CM microC or microPH treatment. ***H***, Representative confocal images of neuroblast cell populations AB, CD, EF, and total neuroblasts. Neuroblast cells are labeled with DCX (green). ***I***, Density of neuroblast types AB, CD, and EF. ***J***, Proliferating neuroblasts (BrdU^+^, DCX^+^) density after treatment with CM microC or microPH. ***K***, Experimental design used for the administration of CM microC or microPH by osmotic pumps to 2-month-old fms-EGFP mice. ***L***, Representative confocal images of BrdU^+^ cells in the dentate gyrus. ***M***, BrdU^+^ cell density after CM microC or microPH treatment. ***N***, Apoptotic cell density after CM microC or microPH treatment. ***O***, Representative confocal images of neuroblasts labeled with DCX (green). ***P***, Density of total number of neuroblasts in Sections 1–3 closest to the injection site, and 4–6 further away. ***Q***, Representative confocal images of a newborn neuron labeled with BrdU (yellow) and NeuN (magenta). ***R***, Density of neuroblast types AB, CD, EF, and total neuroblasts. ***S***, New neurons (NeuN^+^, BrdU^+^) density after CM microC or microPH treatment. Scale bars: ***B***, ***L***, ***E***, ***O***, ***H***, 50 μm (insert in ***H***, 20 μm); ***Q***, 20 μm (insert, 10 μm); ***B***, ***L***, ***E***, ***O***, ***H***, *z* = 12 μm; ***Q***, *z* = 6 μm. *N* = 7–10 mice (***B***–***J***), *N* = 5–11 mice (***L***–***S***). Error bars represent mean ± SEM. #*p* = 0.0649, *p < 0.05, **p < 0.01, ***p < 0.001 by Student's *t* test. Values of statistics used are shown in Table 9. ns, not significant.

To then confirm the neurogenic modulatory role of the phagocytosis secretome on adult hippocampal neurogenesis *in vivo*, we injected CM microC and microPH into the hippocampus of 2-month-old fms-EGFP mice for 6 d using osmotic minipumps. After this period, BrdU was administered to track proliferating cells and mice were killed 2 h later (Fig. 14*A*).

We observed no differences in the density of total BrdU^+^ proliferative cells in CM microPH treated mice compared to CM microC treatment (Fig. 14*B*,*C*). Importantly, CM microPH did not induce apoptosis *in vivo* (Fig. 14*D*). In addition, there was a trend toward decreased density of proliferating BrdU^+^ rNSCs in mice treated with CM microPH compared to CM microC (*p* = 0.0649; Fig. 14*E–G*). Moreover, the density of DCX^+^ neuroblasts, the proportion of the different neuroblast subpopulations (AB, CD, and EF), and neuroblast proliferation did not differ between CM microC and microPH (Fig. 14*H–J*).

Because we observed a declining trend for rNSCs in the presence of CM microPH after 6 d, we hypothesized that the induced alterations would accumulate over time. Therefore, we performed a long-term experiment in which mice were treated with CM microC and microPH through osmotic minipumps for 6 d, followed by BrdU administration, and killed 28 d later to allow differentiation of the labeled cells (Fig. 14*K*). The density of BrdU^+^ cells in mice treated with CM microPH showed a significant decrease compared to CM microC (Fig. 14*L*,*M*). Importantly, CM microPH did not induce apoptosis *in vivo* (Fig. 14*N*). We then quantified neuroblasts and newborn neurons (NeuN^+^, BrdU^+^; Fig. 14*O–S*). In all experiments in Figure 14, because the conditioned media diffused over the 6 d infusion period, we found a stronger effect closer to the injection site (Fig. 14*P*). Thus, for this set of experiments, only the three tissue slices of the sectioning series most proximal to the injection site were quantified. We found a significant reduction in the intermediate (CD) and most mature (EF) neuroblast subpopulations in mice treated with CM microPH compared to CM microC (Fig. 14*R*). Finally, the density of newborn neurons was also reduced in CM microPH compared to CM microC (Fig. 14*S*). In summary, we found a trend toward fewer proliferative stem cells at 2 h after BrdU, and reduced number of mature neuroblasts and newborn neurons after 28 d, suggesting that the acute (24 h) phagocytic microglia secretome limits neurogenesis via the reduction in the production of neuronal-committed cells.

## Discussion

In this paper, we provide evidence that microglia modulate adult hippocampal neurogenesis through the secretome associated with phagocytosis of apoptotic newborn cells, based on the following major findings. First, adult hippocampal neurogenesis was reduced in two KO models with chronic microglial phagocytosis impairment. Second, neurogenesis was transiently increased in an acute model of phagocytosis impairment. Third, transcriptomic analysis *in vitro* revealed that phagocytosis triggered an expression change in a panoply of neurogenesis-related genes in microglia, strongly suggesting a coordinated neurogenic modulatory program that encompasses up to 224 heterologous genes previously shown to modulate neurogenesis, including peptides, trophic factors, matrix metalloproteases, and cytokines. Fourth, the secretome of phagocytic microglia drove NPCs differentiation toward a bipolar phenotype of astrocytic lineage *in vitro*, characterized by high expression of astrocytic markers such as nestin, GFAP and S100β; calcium responses to ATP and other stimuli; and high levels of phosphorylation of SMAD. Finally, the secretome of phagocytic microglia reduced the most mature neuroblast subpopulation at 28 d *in vivo*. Hence, we present evidence that microglial phagocytosis is a pivotal mechanism for the maintenance of homeostasis in the adult neurogenic cascade.

### Microglia control the long-term homeostasis of the adult hippocampal neurogenic cascade

The accurate homeostasis of stem cell niches is crucial for their long-term maintenance, as disruption of the equilibrium between quiescence and proliferation leads to early exhaustion (Santos et al., 2018). In the adult hippocampal neurogenic niche, the mechanisms described to maintain homeostasis rely on the quiescence of rNSCs (Encinas et al., 2011), a feature that prevents an early exhaustion of the niche (Sierra et al., 2015). Herein we focus on the unexplored role of resident immune cells, microglia, which engulf newborn cells that undergo apoptosis (Sierra et al., 2010). Immune cells are increasingly recognized to participate in stem cell niches, and macrophages have been recently shown to promote erythroblast production in the bone marrow (Chow et al., 2013), and to be required for ductal morphogenesis in the mammary gland (Chakrabarti et al., 2018). We found that chronic disruption of microglial phagocytosis impairs neurogenesis using two constitutive KO mice models for receptors P2Y12, and MerTK/Axl, which participate in different stages of phagocytosis (Scott et al., 2001; Elliott et al., 2009). Nonetheless, it is important to note that these receptors regulate multiple features of microglial physiology, and that MerTK and Axl are also expressed in peripheral macrophages (Rothlin and Lemke, 2010). In addition, microglial phagocytosis impairment leads to the accumulation of non-removed apoptotic cells, which may also affect neurogenesis directly through the release of toxic intracellular contents. However, the similar reduction in adult neurogenesis in the two models strongly supports the key role of microglial phagocytosis.

**Figure 15.**
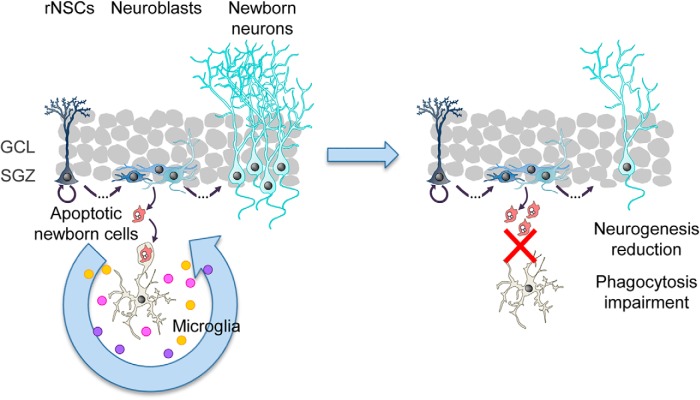
Microglia provides a feedback loop that controls neurogenesis through the phagocytosis-secretome.

In contrast to the effect of constitutive phagocytosis impairment, acute phagocytosis impairment by inducible depletion of MerTK resulted in a transient increase in early neuroblasts that was compensated at later time points. Several effects may explain the differences between the constitutive MerTK/Axl KO and the inducible MerTK KO mice (i.e., double KO vs single KO; body-wide in all TAM-expressing cells vs microglial-specific; and knock out from embryonic development vs only in selected cells in the adult). Nonetheless, the transient increase in early neuroblasts after acute impairment of phagocytosis in the iKO MerTK model, together with the chronic reduction of neurogenesis in the P2Y12 and MertK/Axl constitutive KO models, and the reduction in neurogenesis in mice treated with the phagocytic microglia conditioned media, suggest that the microglial phagocytosis of newborn cells participates in a feedback loop that maintains the homeostasis of adult hippocampal neurogenesis (Fig. 15).

### Microglia regulates neurogenesis through the phagocytosis secretome

We found that phagocytosis of apoptotic cells induced a neurogenic modulatory phenotype in microglia that was mostly related to their secretome, as the majority of the modulatory genes encoded secreted proteins, including neuropeptides such as VGF, and growth factors such as VEGF and FGF2, some of which have already been described to participate in the microglial regulation of neurogenesis (Kreisel et al., 2019). In addition, the microglial secretome may contain metabolites, miRNAs and extracellular vesicles, which may also alter neurogenesis (Rodríguez-Iglesias et al., 2019). When administered *in vivo*, the acute secretome of phagocytic microglia inhibited hippocampal neurogenesis, as it had an early tendency to decrease rNSCs proliferation that was later followed by a reduction in mature neuroblasts. The effect was similar on isolated NPCs, as we found a decreased production of neuroblasts. However, this effect was unlikely related to an active promotion of apoptosis, which was not detected *in vivo*. In agreement, the *in vitro* transcriptomic analysis did not reveal significant increases in heterologous proapoptotic genes, and cell death was not observed in the late survival/differentiation assays. Nonetheless, many early NPCs did die upon culture with the early phagocytic microglia conditioned media, an effect that may be attributed to the lack of key survival factors, possibly metabolites consumed by phagocytic microglia. Apoptosis is common in these early cultures and has been linked to the stress associated with differentiation upon growth factor withdrawal (Babu et al., 2011). Overall, these results suggest that the secretome of phagocytic microglia modulates neurogenesis by acting not on the survival but on the differentiation of neural-committed cells.

The reduced neuronal differentiation induced by the secretome of phagocytic microglia was unlikely related to an enhancement of gliogenesis. *In vivo* no changes in the production of newborn astrocytes were observed in the neurogenic cascade, although on isolated NPCs the phagocytic microglial secretome gave rise to astrocyte-committed cells with a bipolar phenotype, reminiscent of radial glia (Encinas and Enikolopov, 2008). These cells presented several astrocytic features, including the expression of GFAP and S100β (Raponi et al., 2007; Encinas and Enikolopov, 2008); intracellular calcium response to ATP (De Melo Reis et al., 2011) and high phosphorylation of SMAD, which interacts with TGFβ to give rise to astrocytes/radial glia (Stipursky and Gomes, 2007). Nonetheless, our transcriptional assay did not show heterologous 'gliogenic' genes in phagocytic microglia, suggesting that rather than actively promoting astrogenesis, the acute phagocytosis secretome indirectly promote the default astrocytic lineage (Miller and Gauthier, 2007) by restricting the neuronal lineage.

### Cytokines are unrelated to the effect of phagocytosis secretome on neurogenesis

Several cytokines were also expressed by phagocytic microglia, such as IL-1β, IL-6, and TNFα, which have been reported to decrease survival of neuroprogenitors *in vitro* (IL-1β, TNFα) and *in vivo* (IL-6; Breton and Mao-Draayer, 2011), inhibiting adult neurogenesis. This cytokine expression profile of phagocytic microglia holds some parallelism to the proinflammatory profile triggered upon inflammation, a process that impairs neurogenesis (Ekdahl et al., 2003; Monje et al., 2003). However, in our hands the inflammatory microglia secretome did not trigger a reduction in the survival of NPCs. Furthermore, we found that treatment with neither LPS nor the secretome of LPS-stimulated microglia reduced the production of neuroblasts *in vitro*, suggesting that inflammation is not as detrimental for neurogenesis as previously stated (Ekdahl et al., 2003; Monje et al., 2003) and that cytokines were not responsible for the effects of phagocytic microglial secretome on neural-committed cells. In addition, the neurogenic modulatory program initiated by phagocytosis encompassed genes involved in matrix remodeling (matrix metalloproteases) and membrane ligands (Jag1, ligand for Notch receptor), suggesting that the observed direct contact between microglia and rNSCs/neuroblasts (Sierra et al., 2010) may also participate in shaping the neurogenic niche through participating in the local control of neuroblast differentiation, survival and synaptic integration (Rodríguez-Iglesias et al., 2019).

### Phagocytosis reprograms microglia

Finally, we here show that phagocytosis is not simply a terminal process designed to eliminate debris. In fact, in peripheral macrophages engulfment and degradation result in epigenetic, metabolic and functional reprogramming, a process named ′trained immunity′ (Bekkering et al., 2018). Similarly, we here show that in microglia phagocytosis of apoptotic cells triggers a coordinated transcriptional program that involves key chromatin remodeling and metabolic genes, suggesting a long-term reprogramming that may affect multiple microglial functions, from spine surveillance to inflammation. Whether these changes are triggered by the recognition of find-me and “eat-me” surface receptors, or by downstream steps in the phagocytic process of apoptotic cells, remains to be determined. Apoptosis is a widespread phenomenon in neurodegenerative diseases (Abiega et al., 2016) and we speculate that phagocytosis of cell debris and the subsequent alteration of the secretome, as well as other potential functions, may be a key to understanding how microglia impacts surrounding surviving neurons. Similarly, during aging (Pluvinage et al., 2019) or in diseases in which microglial phagocytosis is impaired, such as epilepsy (Abiega et al., 2016), the beneficial effects of promoting engulfment/degradation of cell debris may go beyond merely removing corpses to actively promote regeneration.

In summary, in this paper, we provide strong evidence that phagocytic microglia are a central mechanism to control the homeostasis of the adult hippocampal neurogenic cascade by acutely providing a negative feedback loop via their secretome. This 'brake′ is necessary for the long-term maintenance of the neurogenic cascade, since neurogenesis is transiently increased when phagocytosis is acutely blocked, but is disrupted when microglial phagocytosis is chronically impaired, as observed in genetically deficient mice for P2Y12 and MerTK/Axl. Importantly, the link between the proliferation of newborn cells and apoptosis was already suggested to be necessary for the correct learning and memory of the adult brain (Dupret et al., 2007), and our data here points toward microglial phagocytosis of apoptotic cells as the connecting mechanism. As apoptosis is closely related to neural stem cell proliferation, our data suggest that microglial phagocytosis may also shape other developmental and adult neurogenesis sites, such as the SVZ (Cunningham et al., 2013). In addition, phagocytosis of newborn cells has also been recently shown to play a role in sculpting sex differences in the developing amygdala (VanRyzin et al., 2019). While previous work has suggested a largely detrimental effect of microglia on hippocampal neurogenesis (Valero et al., 2016), our data are in agreement with recent evidences supporting the essential role of macrophages and other immune cells in remodeling stem cells niches (Naik et al., 2018).
